# Multidimensional clustering of clinical, metabolic, and ultrasound features for 30-day mortality risk stratification in ischemic stroke patients with carotid atherosclerotic plaque

**DOI:** 10.3389/fneur.2026.1878670

**Published:** 2026-09-08

**Authors:** Liyuan Fu, Shuxia Huang, Yongjie Zou, Lei Li

**Affiliations:** 1 Department of Ultrasound, The Eighth Medical Center of PLA General Hospital, Beijing, China; 2 The Second People's Hospital of Lishui City, Lishui, China; 3 Department of Neurosurgery, The 908th Hospital of the Joint Logistics Support Force, Nanchang, China

**Keywords:** carotid plaque, ischemic stroke, preliminary phenotypic stratification of short-term risk, prognosis, UMAP, unsupervised clustering

## Abstract

**Background:**

Carotid atherosclerotic plaques are a major cause of ischemic stroke, but stenosis-based assessment does not fully capture plaque heterogeneity or prognostic risk. This study aimed to explore data-driven patient phenotypes and preliminary risk stratification by integrating clinical, metabolic, and carotid ultrasound plaque features using unsupervised clustering.

**Methods:**

Ischemic stroke patients admitted within 72 h of onset were retrospectively enrolled from four centers. Baseline demographics, National Institutes of Health Stroke Scale scores, laboratory indicators, carotid ultrasound plaque characteristics (for example, stenosis rate, Plaque Reporting and Data System grade), and 30-day mortality were extracted. After preprocessing and standardization, *k*-means clustering was applied to baseline features only; 30-day mortality was reserved for post-clustering outcome comparison. The optimal number of clusters was determined using the elbow method and silhouette score, and Uniform Manifold Approximation and Projection was used solely for two-dimensional visualization. Univariable and multivariable logistic regression analyses evaluated whether cluster membership was independently associated with 30-day mortality after adjustment for major clinical, laboratory, and plaque-related confounders.

**Results:**

Among 3,764 patients, five exploratory clusters were identified, showing marked heterogeneity in age, neurological severity, metabolic profile, and plaque characteristics. Cluster 0 (29.2%) comprised the youngest patients, with the lowest National Institutes of Health Stroke Scale scores, smallest plaques, and lowest 30-day mortality (2.9%), whereas Cluster 4 (13.6%) had the highest stenosis rates, more unstable plaque features, pronounced metabolic abnormalities, and the highest mortality (11.2%). Overall mortality differences were significant (chi-squared = 12.36, *p* = 0.015). In multivariable analysis, cluster membership remained independently associated with 30-day mortality: compared with Cluster 0, Cluster 2, Cluster 3, and Cluster 4 showed progressively increased adjusted odds of death.

**Conclusion:**

Integrating multidimensional clinical, metabolic, and ultrasound features with *k*-means clustering can identify exploratory patient phenotypes and reveal substantial heterogeneity in short-term risk among ischemic stroke patients with carotid plaques. High-risk clusters with more severe plaque and metabolic abnormalities suggest potential phenotypic heterogeneity in short-term risk, but these findings should be regarded as hypothesis-generating and require external validation before any clinical application can be considered. The retrospective design, reliance on 30-day mortality alone, and limited clustering stability assessment are important limitations; prospective studies with longer follow-up and external validation are needed to confirm clinical utility.

## Background

Carotid atherosclerotic plaques remain a major risk factor for ischemic stroke. Globally, stroke persists as a leading cause of death and disability, and patients with carotid plaques face a high risk of secondary stroke (1). The key to preventing and managing this condition lies in the accurate assessment of plaque risk. However, traditional stenosis-based approaches have obvious limitations, failing to fully capture the complex relationship between plaque heterogeneity and clinical outcomes. Therefore, this study aims to overcome current assessment limitations by employing multi-dimensional data integration and machine learning techniques to characterize clinical subtypes of carotid plaques, thereby providing a more robust basis for precise preliminary phenotypic stratification of short-term risk and individualized intervention.

The association between carotid plaques and stroke has been well established by multiple large cohort studies. Approximately 20% of ischemic strokes are directly attributable to carotid plaques. Patients with carotid stenosis ≥50% have a significantly increased stroke risk (2) and are classified as the TOAST classification of Large-artery atherosclerosis (LAA) type (3). With the aging of the population, the prevalence of carotid plaques in Individuals over 60 years old is as high as 30–40%, posing a major public health challenge (4). The Framingham Heart Study, MESA study, and others have shown that traditional risk factors such as age, hypertension, hyperlipidemia, and diabetes are significantly associated with carotid intima-media thickness (IMT) and plaque burden, accelerating the progression of atherosclerosis and contributing acute cardiovascular events (5). In China, regional differences in stroke distribution further suggest that environmental, genetic, and lifestyle factors indirectly affect stroke risk by influencing the progression of carotid plaques (6).

While traditional paradigms emphasize that carotid stenosis degree is the core indicator of stroke risk, recent studies have confirmed that the biological properties of plaques (such as stability, inflammatory status, and lipid composition) are more critical predictors. Patients with unstable plaques, characterized by a large lipid necrotic core, thin fibrous cap, ulceration, or intraplaque hemorrhage have a 3- to 5-fold higher stroke risk than those with stable plaques (7). Furthermore, plaque instability is associated with poorer prognosis and higher mortality rates (8). In addition, metabolic indicators such as low-density lipoprotein cholesterol and inflammatory markers such as C-reactive protein (CRP) are closely related to plaque progression and inflammatory activity, reflecting pathological changes in the local microenvironment of plaques. For example, elevated CRP levels are associated with inflammatory infiltration in plaques, while elevated low-density lipoprotein accelerates lipid deposition and fibrous cap degradation, both significantly increasing stroke risk (9) and mortality.

Currently, clinically used preliminary phenotypic stratification of short-term risk methods are mainly based on imaging stenosis rates such as the North American Symptomatic Carotid Endarterectomy Trial [NASCET] criteria combined with traditional risk factors such as hypertension and diabetes. However, approximately 30% of strokes occur in patients with stenosis <50%, indicating that the existing system is unable to identify the “low stenosis but high risk” population (10). In addition, the limitations of traditional methods also do not adequately capture dynamic plaque changes, such as progression rate and individual metabolic heterogeneity, leading to insufficient identification of potential high-risk subgroups. For example, Blinc et al. (10) reported that carotid plaque burden and progression rate have better predictive value for cardiovascular events than stenosis degree alone, and plaque echo characteristics such as hypoechogenicity indicating lipid core are more closely related to plaque vulnerability. Moreover, patients show significant differences in response to pharmacotherapy especially anti-lipid, further highlighting the need for precise stratification.

To overcome the limitations of traditional stenosis indicators, institutions such as the American College of Radiology (ACR) have launched Plaque-RADS (Carotid Plaque Reporting and Data System). This standardized classification tool utilizes multi-modal imaging (ultrasound, CTA, MRI) to integrate plaque morphology, composition, and stenosis degree, significantly improving the accuracy of risk assessment (11, 24). Plaque-RADS classifies carotid plaques into grades 1–4 with further subgroup divisions. Studies have confirmed that combining stenosis degree with Plaque-RADS scores can significantly improve the accuracy of preliminary phenotypic stratification of short-term risk, especially in patients with mild to moderate stenosis (<70%): compared with those with Plaque-RADS <3, patients with scores ≥3 have significantly shorter disease-free survival (DFS) and recurrence-free survival (RFS); the net reclassification improvement (NRI) reaches 63.8 and 47.8% in asymptomatic and stroke-history populations, respectively, effectively identifying high-risk individuals that may be missed by traditional methods (12). However, the differentiation between Plaque-RADS grade 3 subgroups (e.g., 3b thin-cap fibroatheromas) and grade 4 high-risk plaques still relies on multi-modal imaging like MRI enhanced scanning to identify IPH and comprehensive clinical assessment. While MRI and other examinations are expensive and have long waiting times, making them difficult to popularize and perform well in most regions.

Machine learning algorithms have been widely used in preliminary phenotypic stratification of short-term risk and prediction of neurological diseases (13, 25). Most studies employ supervised learning methods, showing great potential in carotid plaque risk assessment. For example, Fang et al. (14) integrated ultrasound and clinical factors to construct a random forest model for predicting plaque vulnerability in gout patients, with a C-index as high as 0.997. This not only significantly improved accuracy but also revealed risk patterns not identified by traditional methods, such as tophi (OR = 1.760), power Doppler signal grade (Grade 3: OR = 1.890), and annual gout attack frequency (OR = 1.524), confirming the effectiveness of supervised learning in risk assessment combining clinical indicators (14).

Unsupervised learning methods are currently mainly applied in medical image processing. In carotid plaque analysis, unsupervised clustering algorithms like *k*-means combined with dimensionality reduction techniques of UMAP is often used for ultrasound image analysis (15). However, the application of unsupervised learning combined with clinical indicators is relatively limited, and its potential in integrating clinical and image data for carotid plaque risk assessment has not been fully exploited.

A study by Li et al. (16) combined CTA radiomics with clinical factors to construct a composite model for ischemic stroke risk assessment. The model achieved an AUC of 0.878 in the training set, significantly outperforming the clinical model alone (0.766) or the radiomics model alone (0.766), fully demonstrating the added value of multi-modal data integration in carotid plaque-related risk assessment. This study indicates that comprehensive use of clinical and imaging indicators can more accurately assess carotid plaque-related risks, providing a more reliable basis for clinical decision-making.

Building on previous research, the present study aims to address the core challenges of carotid plaque preliminary phenotypic stratification of short-term risk through systematic integration of multidimensional data and machine learning methods by employing a large-sample approach encompassing diverse ages and genders while collecting a comprehensive set of metabolic indicators including inflammatory factors, blood lipids and electrolytes alongside ultrasonic plaque features such as IMT, Plaque-RADS grade, echo intensity and stenosis rate to ensure data comprehensiveness.

By correlating metabolic characteristics, traditional risk factors, Plaque-RADS imaging classification, and plaque-related ultrasound features within the same cohort, this study seeks to move beyond the limitations of relying solely on stenosis or morphology and to provide a more refined, data-driven framework for carotid plaque preliminary phenotypic stratification of short-term risk.

The research combines *k*-means clustering with UMAP dimensionality reduction to reduce the subjectivity of traditional stratification and identify clinical subgroups of carotid plaques through data-driven means such as metabolic-driven high-risk groups and inflammatory progressive groups. By uncovering subgroup-specific risk patterns and phenotypic characteristics, this study provides a data-driven basis for refined preliminary phenotypic stratification of short-term risk of carotid plaque patients. High-risk exploratory phenotypes identified in this clustering analysis may warrant closer clinical monitoring and careful application of guideline-based secondary prevention, but the present study does not support direct individualized treatment decisions based solely on cluster assignment.

## Materials and methods

### Study subjects

#### Inclusion criteria

A total of 3,764 cases of stroke secondary to carotid artery plaques between 2017.3.1 and 2022.12.31 were collected from four centers: the 8th Medical Center of the General Hospital of the Joint Logistics Support Force, the 305th Hospital of the Chinese People’s Liberation Army, the Second People’s Hospital of Lishui City, and the 908th Hospital of the Joint Logistics Support Force. We have got the Ethics approval from the Ethics Committee of the Eighth Medical Center of PLA General Hospital and then collected the database.

The inclusion criteria are as follows: (1) patients diagnosed with ischemic stroke by cranial CT, MRI, or other imaging examinations; (2) within 72 h from onset to enrollment; (3) aged between 18 and 80 years.

#### Exclusion criteria

The Exclusion Criteria are as follows: (1) complicated with end-stage liver or kidney failure (Child-Pugh class C or glomerular filtration rate <15 mL/min/1.73m^2^); (2) diagnosed with malignant tumors (except low-progressive tumors such as non-cutaneous basal cell carcinoma) in progressive or end-stage; (3) suffering from mental diseases (such as schizophrenia, severe cognitive impairment, etc.) and unable to cooperate with the study process; (4) permanent atrial fibrillation or other reason of confirmed cardioembolic stroke (5) missing key clinical data (such as onset time, imaging results, etc.), making it impossible to fully assess the condition.

### Selecting procedure

After extracting 13,355 stroke patients from the four centers between March 1, 2017 and December 31, 2022, we first applied the predefined inclusion criteria. A total of 8,523 patients met the inclusion criteria, including confirmed ischemic stroke by imaging, onset-to-enrollment time within 72 h, and age between 18 and 80 years.

Subsequently, exclusion criteria were applied sequentially. Patients were excluded if they had end-stage liver or renal failure (*n* = 351), progressive or end-stage malignancy (*n* = 488), severe psychiatric disorders or inability to cooperate with the study (*n* = 1,201), confirmed cardioembolic stroke such as permanent atrial fibrillation (*n* = 58), or missing key clinical data (*n* = 3,149). After these exclusions, a total of 3,764 patients were included in the final analysis.

### Key data extraction

Data extraction in this study covers four dimensions: demographics, clinical scores, laboratory tests, and ultrasonic plaque features. Variables used for clustering were restricted to baseline demographic, clinical, laboratory, and carotid ultrasound features collected at admission. Thirty-day mortality was not included in the clustering inputs and was reserved for subsequent subgroup outcome comparison. The specific indicators and extraction criteria are as follows:

#### Demographics and clinical scores

The following basic information was extracted from the study database: demographic indicators include gender (categorical variable: male/female) and age (continuous variable, unit: years). Clinical scores include the National Institutes of Health Stroke Scale score (NIHSS, continuous variable, range 0–42 points, used to assess stroke severity) NIHSS scoring at admission [median 6 h (IQR 4–12 h) post-onset], and fasting venous blood collection within 24 h of admission.

#### Laboratory indicators

Laboratory data were extracted from the lab_tests table, with records containing more than 30% missing values excluded. Specific indicators are classified into subgroups as follows:

Inflammatory and hematological indicators include white blood cell count (WBC, ×10^9^/L), red blood cell count (RBC, ×10^12^/L), platelet count (PLT, ×10^9^/L), C-reactive protein (CRP, mg/L), lactate dehydrogenase (LDH, U/L), α-hydroxybutyrate dehydrogenase (HBDH, U/L), creatine kinase (CK, U/L), creatine kinase-MB (CK_MB, U/L), and ischemia-modified albumin (IMA, U/mL).

Coagulation function indicators include international normalized ratio (INR, unitless), prothrombin time (PT, s), activated partial thromboplastin time (aPPT, s), thrombin time (TT, s), D-dimer (D_dimer, mg/L FEU), fibrinogen (fibrinogen, g/L), and fibrin degradation products (FDP, mg/L).

Biochemical and metabolic indicators include glycated hemoglobin (HbA1c, %), triglycerides (TG, mmol/L), low-density lipoprotein cholesterol (LDL, mmol/L), high-density lipoprotein cholesterol (HDL, mmol/L), plasma viscosity (plasma_viscosity, mPa·s), lactic acid (lactate, mmol/L), sodium (Na, mmol/L), potassium (K, mmol/L), chlorine (Cl, mmol/L), calcium (Ca, mmol/L), and phosphorus (P, mmol/L).

Blood gas analysis indicators include partial pressure of carbon dioxide (PaCO_2_, mmHg), partial pressure of oxygen (PaO_2_, mmHg), pH value (pH, unitless), bicarbonate (HCO_3_, mmol/L), total carbon dioxide (tCO_2_, mmol/L), and anion gap (anion_gap, mmol/L).

Liver and kidney function indicators include aspartate transaminase (AST, U/L), alanine transaminase (ALT, U/L), creatinine (creatinine, μmol/L), urea (urea, mmol/L), bilirubin (bilirubin, μmol/L), albumin (albumin, g/L), and blood uric acid (BUA, μmol/L).

#### Carotid ultrasound plaque features

Carotid ultrasound was performed at a median of 12 h post-stroke onset, the following features were extracted from the ultrasound_data table and ultrasound diagnostic report texts with details supplemented based on plaque pathological characteristics:

Plaque location records the anatomical location of the plaque, including carotid bifurcation, distal common carotid artery, origin of internal carotid artery, and other sites. Plaques at the carotid bifurcation, distal common carotid artery, and origin of internal carotid artery are more prone to instability due to hemodynamic effects and need to be highlighted.

Maximum plaque thickness measures the maximum distance of the plaque perpendicular to the vessel wall, which is a key size indicator for assessing plaque stability (more clinically significant than length). For example, if the plaque size is recorded as 2.0 × 3.5 mm, 2.0 mm is the thickness value; generally, thickness >2.0 mm suggests an increased risk of instability.

Maximum plaque length measures the longest diameter of the plaque parallel to the vessel wall, serving as an auxiliary indicator to reflect plaque burden, and combined with thickness to assess the overall size of the plaque.

Echo pattern is divided into hypoechoic, isoechoic, hyperechoic, and mixed echo. Hypoechoic (rich in lipid or necrotic core) and mixed echo plaques containing hypoechoic components are defined as unstable echoes (unstable_echo = “yes”), while isoechoic and hyperechoic plaques are defined as stable echoes (unstable_echo = “no”).

Irregular shape is judged based on the description in the ultrasound report. If the plaque edge is irregular, lobulated, or contorted, it is recorded as “yes”; such morphology is significantly associated with instability risk.

Surface integrity reflects the state of the fibrous cap, including fibrous cap rupture, ulceration, thrombus attachment, etc. If the report mentions any of the above, it is recorded as “yes”; if the fibrous cap is intact with no ulceration or thrombus, it is recorded as “no.” Surface disintegration is an important predictor of acute plaque events.

Stenosis rate is measured by ultrasound Doppler, calculated as (1 − residual lumen diameter at stenosis/lumen diameter at normal segment) × 100%.

Risk level is determined by ultrasound doctors based on location, thickness, echo, morphology, and surface integrity and extracted from the report. For example, a hypoechoic plaque with incomplete surface at the carotid bifurcation is judged as “high” risk.

Stenosis classification is divided into mild <50%, moderate 50–69%, and severe ≥70%, following the NASCET criteria.

Plaque-RADS score is combined with echo characteristics (e.g., proportion of hypoechogenicity), surface integrity (e.g., ulceration), and stenosis degree. Grade 1 is a stable plaque, and grade 4 is an extremely high-risk plaque (e.g., hypoechoic plaque with ulceration).

For conventional plaque features (location, maximum thickness, maximum length, echo pattern, surface integrity, stenosis rate), initial data were extracted from structured clinical ultrasound reports, and all items were further cross-validated via independent review of original ultrasound images. The extracted data were also cross-validated through text descriptions in ultrasound reports. For example, for the record “A 2.5 × 4.0 mm hypoechoic plaque is visible at the carotid bifurcation, with irregular edges and visible ulceration on the surface,” it should be correspondingly labeled as plaque_location = “carotid bifurcation,” max_thickness = 2.5 mm, echo_pattern = “hypoechoic,” unstable_echo = “yes,” irregular_shape = “yes,” surface_unintegrity = “yes,” etc. All ultrasound examinations were performed by senior sonographers with more than 5 years of experience in vascular ultrasound. For data with ambiguous diagnosis or contradictions in the report, more than 2 professional ultrasound doctors independently reviewed the original ultrasound images, conducted multi-section dynamic playback and measurement for controversial points. Doctors in the group uniformly analyzed and confirmed through case discussion meetings, selected 10–15 representative typical images (including cases with different echo types and extreme thickness values), labeled features in combination with clinical data, formed standardized image analysis reports, and used boxplots for outlier visualization verification of numerical data such as plaque_max_thickness.

Notably, Plaque-RADS scores were not directly extracted from clinical reports. Instead, two trained vascular sonographers independently assigned Plaque-RADS grades for each plaque according to the official classification criteria, based on full review of the original B-mode ultrasound images with comprehensive assessment of echo pattern, plaque morphology, surface integrity, and stenosis degree. Discrepancies between the two reviewers were resolved by consensus discussion with a third senior sonographer. All sonographers involved in image review and Plaque-RADS scoring were blinded to patients’ clinical outcomes, including 30-day mortality, subsequent clustering results, and other prognostic information throughout the assessment process; only baseline ultrasound images and basic patient identifiers were accessible to the reviewers.

Interobserver reliability was formally evaluated in a random 10% subset of the full cohort. For categorical variables, including echo pattern, surface integrity, and Plaque-RADS grade, agreement was quantified using Cohen’s kappa coefficient. For continuous variables, including maximum plaque thickness and stenosis rate, agreement was assessed using the two-way random-effects intraclass correlation coefficient for absolute agreement.

30-day mortality data were obtained by reviewing medical records, death reports and directly phoning the patients’ family members to obtain the mortality field (boolean, ‘true’ indicates death, ‘false’ indicates survival).

Fasting venous blood samples were collected from all patients within 24 h of admission. Serum biochemical parameters, including lipid profiles, glucose, liver and renal function indicators, and inflammatory markers, were analyzed using standard automated biochemical analyzers in the hospital’s central laboratory. All laboratory procedures followed standard operating procedures (SOPs) and underwent daily internal quality control in accordance with ISO 15189 standards to ensure data accuracy and reproducibility.

The four source tables were linked using the unique identifier patient_id, and records meeting the inclusion and exclusion criteria were filtered using SQL WHERE clauses. The screening criteria included age between 18 and 80 years, absence of end-stage malignant tumors, and availability of complete baseline examination data. The filtered data were exported as a CSV file and imported into the study_cohort table within the research_database schema. Continuous variables were standardized using *Z*-scores. Binary variables were encoded as 0/1. Categorical variables were one-hot encoded, and ordinal variables, including Plaque-RADS, were encoded according to their ordered levels. Thirty-day mortality was not used as a clustering feature and was reserved for post-clustering outcome comparison.

### Research methods

Missing data handling Missingness patterns were examined for all baseline variables, and variables with extremely high missingness or poor data quality were excluded a prior from clustering and regression analyses. Mixed-type imputation of remaining missing values was performed using the missForest algorithm (random-forest-based imputation for continuous and categorical data, n_estimators = 100, random_state = 42, max_tree = 100), implemented in Python. The imputation model included all baseline clinical, laboratory, and carotid ultrasound features but excluded the 30-day mortality outcome to avoid leakage. Convergence was assessed using standard out-of-bag prediction error criteria for missForest.

Imputation performance was evaluated by targeted mask-and-impute assessments, in which subsets of observed values for NIHSS and selected laboratory variables were artificially masked, imputed, and compared with the original data using normalized error metrics, and by visual comparison of marginal distributions before and after imputation. Sensitivity analyses were performed by repeating clustering on a NIHSS complete-case subset and refitting the 30-day mortality model with an alternative “median-plus-indicator” imputation strategy; agreement between cluster assignments and model performance and coefficients was compared with those obtained from the missForest-imputed dataset.

#### Determine optimal number of clusters using elbow method and silhouette score

The elbow method and silhouette score method were used to determine the optimal number of clusters. Clustering was performed using baseline clinical, laboratory, and ultrasound features only; 30-day mortality was excluded from the clustering procedure and was used only for post-clustering outcome comparison. In the elbow method, k-means clustering was performed for each number of clusters in the range of 2–10, the sum of squared errors (SSE) was calculated, and a line chart of SSE changes with the number of clusters was drawn. The number of clusters at the inflection point of the line was selected as the candidate optimal value. In the silhouette score method, the average silhouette score corresponding to each number of clusters was also calculated in the range of 2–10, and the number of clusters with the largest silhouette score was selected as the optimal number. The final number of clusters was determined by integrating the results of the two methods.

#### *k*-means clustering and UMAP grouping

Based on the determined optimal number of clusters, the *k*-means clustering algorithm was used to cluster the standardized dataset to obtain cluster labels for each study subject. The UMAP algorithm was used for dimensionality reduction, with the number of neighbors set to 15 and the minimum distance set to 0.1 as the default parameter according to the official UMAP documentation, reducing high-dimensional data to a two-dimensional space. Then, the dimensionally reduced sample points were grouped and visualized according to cluster labels to intuitively display the distribution of different clusters.

### Clustering stability assessment

After determining *k* = 5 using the elbow method and silhouette score, we performed resampling-based clustering stability analyses using the full UMAP-plus-*k*-means pipeline as the reference solution. First, to assess robustness to algorithmic and initialization variability, we reran the complete pipeline—including standardization of baseline features, UMAP dimensionality reduction to 10 components, and k-means clustering at *k* = 5—under 100 different random seeds. For each run, UMAP was refitted with the same parameters but a different random_state, *k*-means was refitted in the embedded space, and agreement with the reference five-cluster solution was quantified using the adjusted Rand index (ARI), a widely used measure of clustering concordance that accounts for chance agreement.

Second, to evaluate robustness with respect to sampling variability, we conducted a bootstrap-style subsampling analysis. For each of 100 iterations, a random 80% subsample of the cohort was drawn, UMAP and *k*-means were refitted on this subsample, and ARI was computed between the subsample clustering and the corresponding subset of labels from the reference full-cohort solution. The distributions of ARI values from repeated-seed and bootstrap/subsampling runs were summarized by their mean, median, and range, and visualized using boxplots.

#### Statistical analysis

Multidimensional descriptive analysis was conducted on valid samples, covering global, Cluster grouping, and Figure feature group-Cluster dual grouping levels. Globally, central tendency, dispersion indicators (mean, SD, median, quartiles, etc.), skewness and kurtosis were calculated to assess data distribution. For Cluster groups, these indicators were computed per variable to clarify inter-group differences. At the dual grouping level, indicators were calculated for each variable in each feature group across Clusters to support subsequent comparisons. All results were exported to Excel for reference. Normality of all variables was tested using Shapiro–Wilk (sample size ≤ 5,000) or Kolmogorov–Smirnov (sample size > 5,000) tests, with *p* > 0.05 as the normality criterion, supplemented by skewness and kurtosis. Tests covered global, Cluster grouping, and dual grouping levels to guide subsequent statistical inference selection. Based on normality results, appropriate inference methods were recommended. For inter-Cluster comparisons, One-way ANOVA with Tukey HSD was used for normal variables, and Kruskal–Wallis *H* test with Dunn method for non-normal ones. Unified standardized visualization was adopted to present data distribution and inter-group differences; Q–Q plots and histograms (with density, mean, and median lines) showed global variable normality, while box plots were drawn by Figure feature group (Cluster as grouping variable) with a unified Set2 palette to display inter-Cluster differences. Missing data were handled by the missForest algorithm for imputation, ensuring the integrity of the dataset while maintaining the reliability of statistical results. For multiple comparisons, Tukey HSD test and Dunn method were adopted for *post-hoc* correction, respectively, for normal and non-normal variables, effectively controlling the type I error rate.

#### Univariable and multivariable regression analysis

To further evaluate the clinical relevance of the clustering results, univariable and multivariable logistic regression analyses were performed to determine whether cluster membership was independently associated with 30-day mortality. Thirty-day mortality was treated as the dependent variable, and odds ratios (ORs) with 95% confidence intervals (CIs) were calculated. In the univariable analysis, each candidate variable, including cluster membership, was tested separately to estimate its crude association with 30-day mortality.

Variables considered clinically relevant or potentially associated with 30-day mortality were then entered into a multivariable logistic regression model. In accordance with the reviewer’s recommendation, the adjusted model included age, sex, baseline NIHSS score, carotid stenosis rate, Plaque-RADS grade, C-reactive protein, LDL-C, HbA1c, renal function indicators, treatment-related variables, and study center effect. Cluster membership was simultaneously included in the model, with Cluster 0 as the reference group, to assess whether the identified subgroups had independent prognostic significance beyond conventional clinical and imaging risk factors. Adjusted ORs, 95% CIs, and *p* values were reported.

Model discrimination was evaluated using the *C*-statistic, and calibration was assessed using the Hosmer–Lemeshow goodness-of-fit test. A two-sided *p* value <0.05 was considered statistically significant.

### Software use

All data analyses, statistical tests, and visualizations were performed using Python programming language (version 3.10), with specific software packages employed for different analytical steps to ensure accuracy and reproducibility. The scikit-learn package (version 1.3.0) was used to implement the k-means clustering algorithm and determine the optimal number of clusters. This clustering analysis was exploratory in nature and intended to generate candidate phenotypes rather than a definitive clinical taxonomy. The umap-learn package (version 0.5.5) was applied for dimensionality reduction and visualization, generating two-dimensional visualization results to verify the separation effect of clustering. For statistical testing, the scipy package (version 1.10.0) and NumPy package (version 1.24.0) were utilized, including normality tests, group comparison tests, and correlation analyses. Data preprocessing, including missing value imputation using the missForest algorithm and variable filtering, was conducted using the pandas package (version 2.0.0) and the missingpy package (version 0.2.0, for implementing missForest imputation). All visualizations (including line charts for the elbow method, Q–Q plots, histograms, and boxplots) were generated using the matplotlib package (version 3.7.0) and seaborn package, with unified font styles, color palettes, and chart parameters to ensure consistency across all figures.

## Research results

### Missing imputation

Across repeated-seed and bootstrap/subsampling runs, ARI values indicated that the five-cluster solution was reproducible under perturbations but not perfectly invariant, and boxplots summarizing these distributions are presented in Supplementary Figures.

In the analysis cohort, NIHSS was missing in 18.3% of patients with carotid ultrasound; variable-level missingness is shown in Supplementary Tables. Mask-and-impute assessments demonstrated a normalized mean squared error of 0.3649 for NIHSS and similarly acceptable error metrics for other key laboratory variables (Supplementary Tables), and marginal distributions before and after imputation were closely aligned (Supplementary Figures).

### Elbow method and silhouette score results

**Figure 1:**
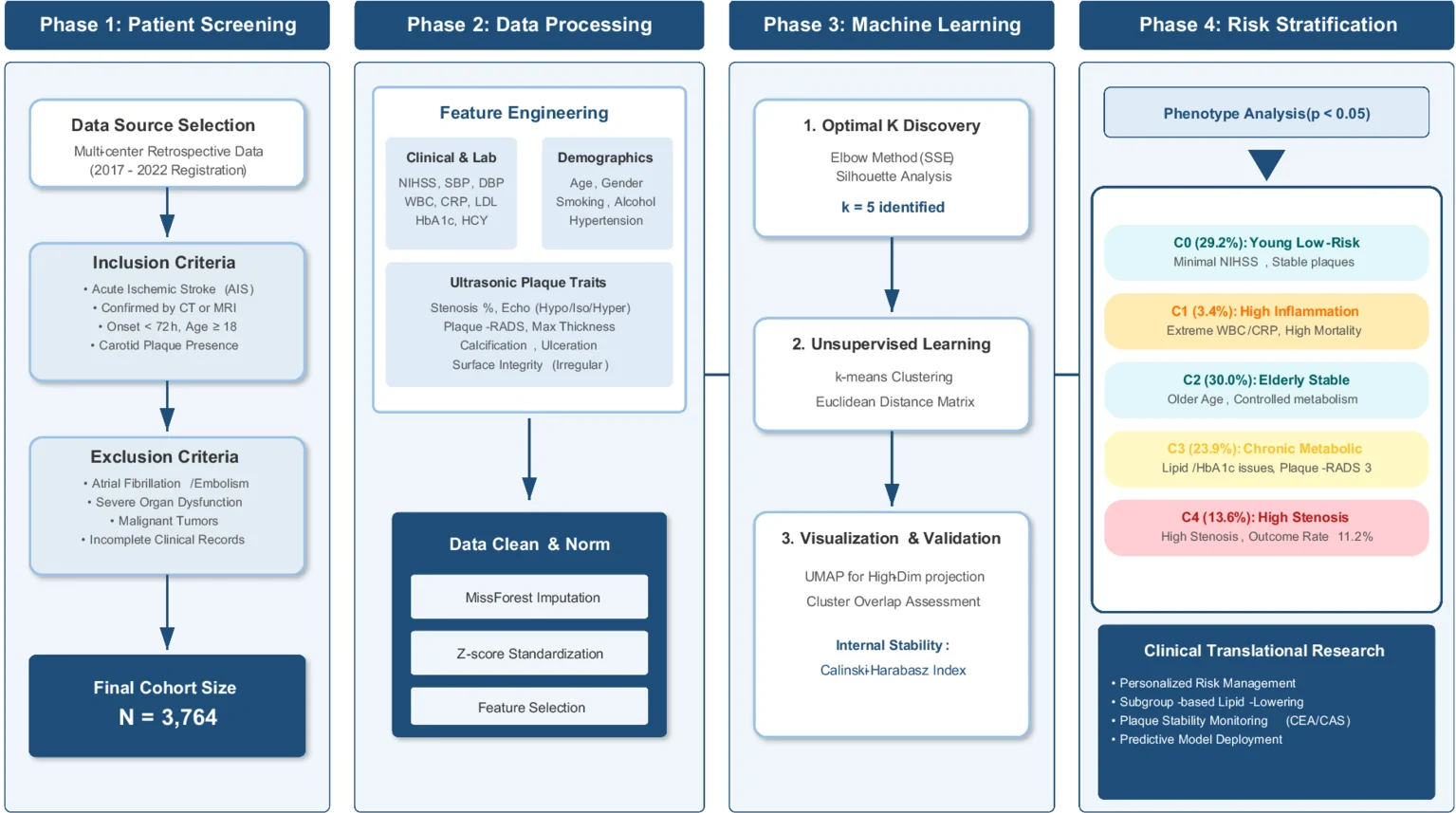
Technical roadmap for risk stratification and subgroup analysis of ischemic stroke patients with carotid plaques via multi-dimensional feature clustering. This flowchart outlines the study workflow: 13,568 patients were screened, with 3,764 eligible participants enrolled after applying inclusion and exclusion criteria. Multi-dimensional data were collected and preprocessed. The optimal cluster number was determined via the elbow method and silhouette score, followed by UMAP dimensionality reduction and *k*-means clustering to identify five distinct subgroups. Inter-subgroup differences were analyzed using appropriate statistical tests, with visualization of key indicators. Finally, subgroup-specific characteristics were summarized to guide personalized clinical interventions.

The line chart drawn by the elbow method showed in Figure 1 that when the number of clusters was 5, the decreasing trend of the sum of squared errors (SSE) slowed down significantly, with an obvious inflection point; the silhouette score method calculated that the average silhouette score was the largest when the number of clusters was 5. Integrating the results of the two methods, the optimal number of clusters was determined to be 5, dividing the study subjects into 5 subgroups (Cluster 0~4).

### *k*-means clustering results

**Figure 2:**
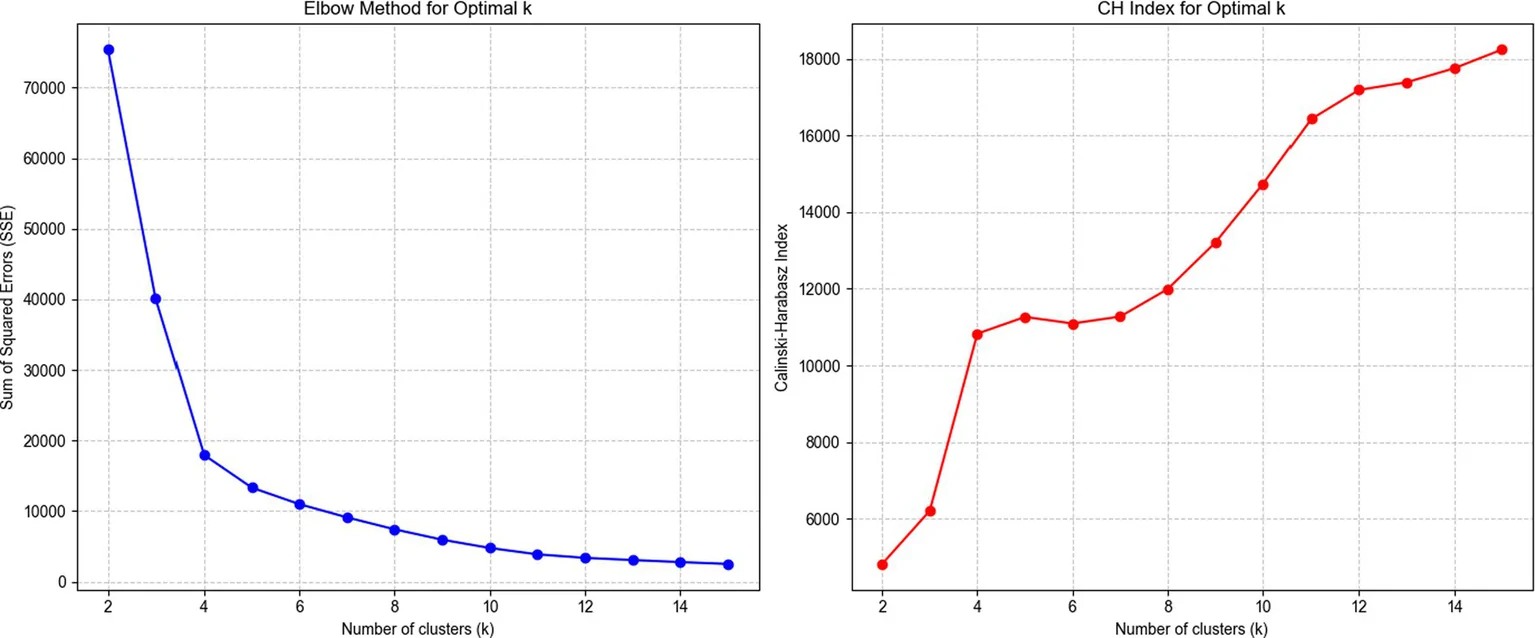
Elbow method and silhouette coefficient curves. This figure displays two clustering optimization curves plotting the number of clusters (*k*-value) against sum of squared errors (SSE) and silhouette coefficient. The elbow point and maximum silhouette coefficient both appear at *k* = 5, indicating that five clusters represent the optimal number for patient stratification in this dataset.

After *k*-means clustering analysis, five subgroups emerged with distinct sample distributions. Cluster 0 included 1,099 participants, accounting for 29.19%. Cluster 1 had 127 participants, making up 3.37%; as the smallest subgroup, which may limit the stability and generalizability of cluster-specific findings; therefore, results for this subgroup should be interpreted cautiously. Cluster 2 was the largest subgroup with 1,130 participants, representing 30.02%. Cluster 3 comprised 897 participants, and Cluster 4 included 511 participants, accounting for 13.57%. The UMAP dimensionality reduction visualization results in Figure 2 showed that the 5 clusters presented a relatively obvious separation trend in the two-dimensional space, providing an intuitive two-dimensional display of the sample distribution across the five clusters.

### Clustering stability analysis

Beyond the initial elbow- and silhouette-based selection of *k* = 5, resampling-based analyses were used to formally evaluate the robustness of the exploratory five-cluster solution. All the Clustering Stability Analysis results is in Supplementary files.

Across 100 repeated runs of the UMAP-plus-*k*-means pipeline with different random seeds, the agreement between each reclustering and the reference five-cluster solution was characterized by ARI, with the distribution of values summarized by its mean, median, and range. Similarly, in 100 bootstrap-style subsampling iterations using 80% of subjects, ARI between each subsample clustering and the corresponding subset of reference labels was computed and summarized in the same way. Overall, the ARI values from both repeated-seed and bootstrap/subsampling analyses indicated moderate stability of the five-cluster solution: the clusters were at least partly reproducible under algorithmic and sampling perturbations, but not perfectly invariant. These findings support the use of the five clusters as hypothesis-generating phenotypes rather than a definitive or highly reproducible clinical taxonomy, and we highlight the limited clustering stability as an important methodological limitation that warrants independent external validation.

### Basic characteristics of clustering results

**Figure 3:**
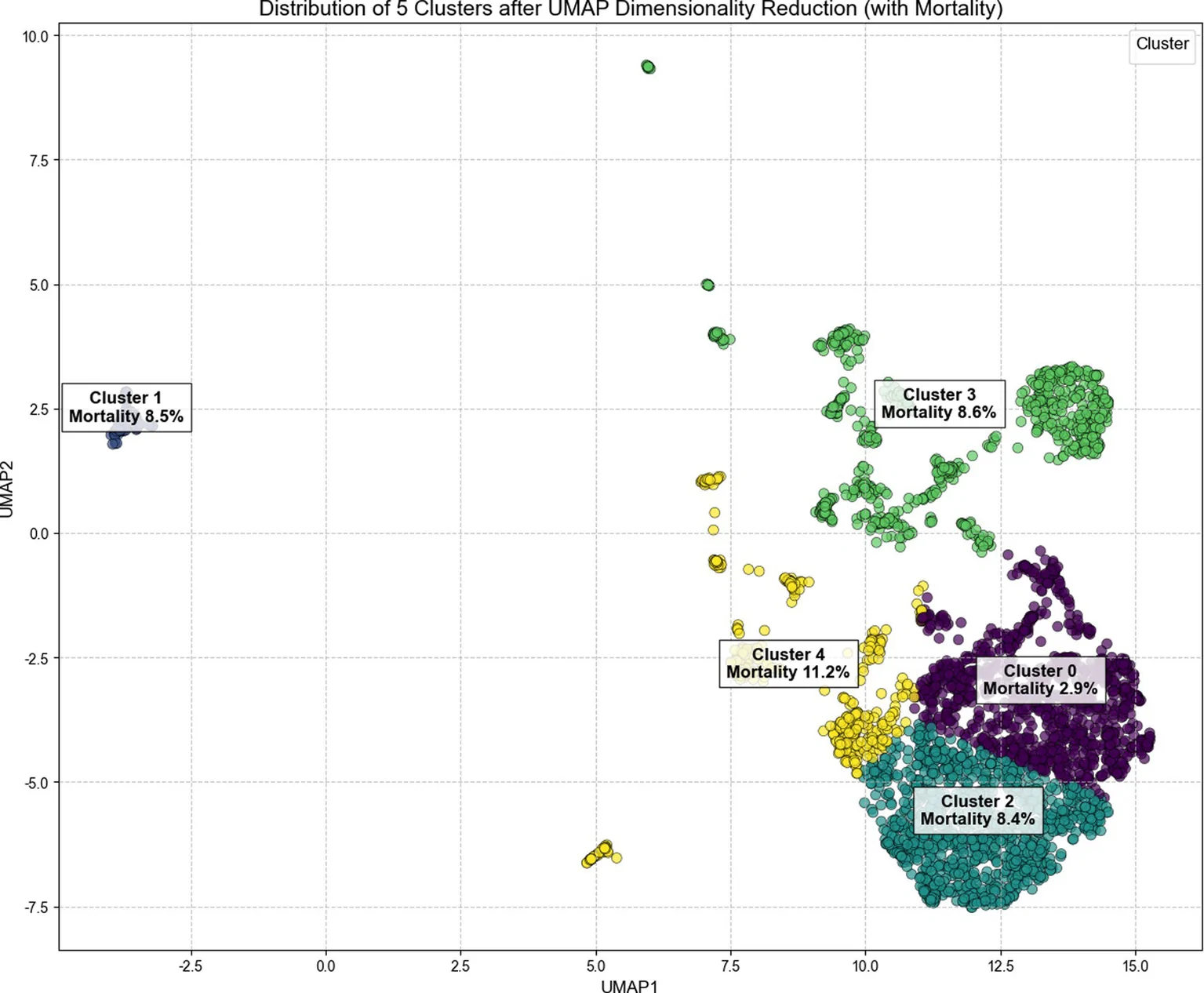
UMAP dimensionality reduction and cluster visualization. This figure presents a two-dimensional visualization of high-dimensional patient data using UMAP algorithm. The five distinct colored clusters (Cluster 0–4) show spatial separation in the reduced dimensional space, demonstrating successful clustering of patients with different clinical profiles into distinct subgroups.

**Figure 4:**
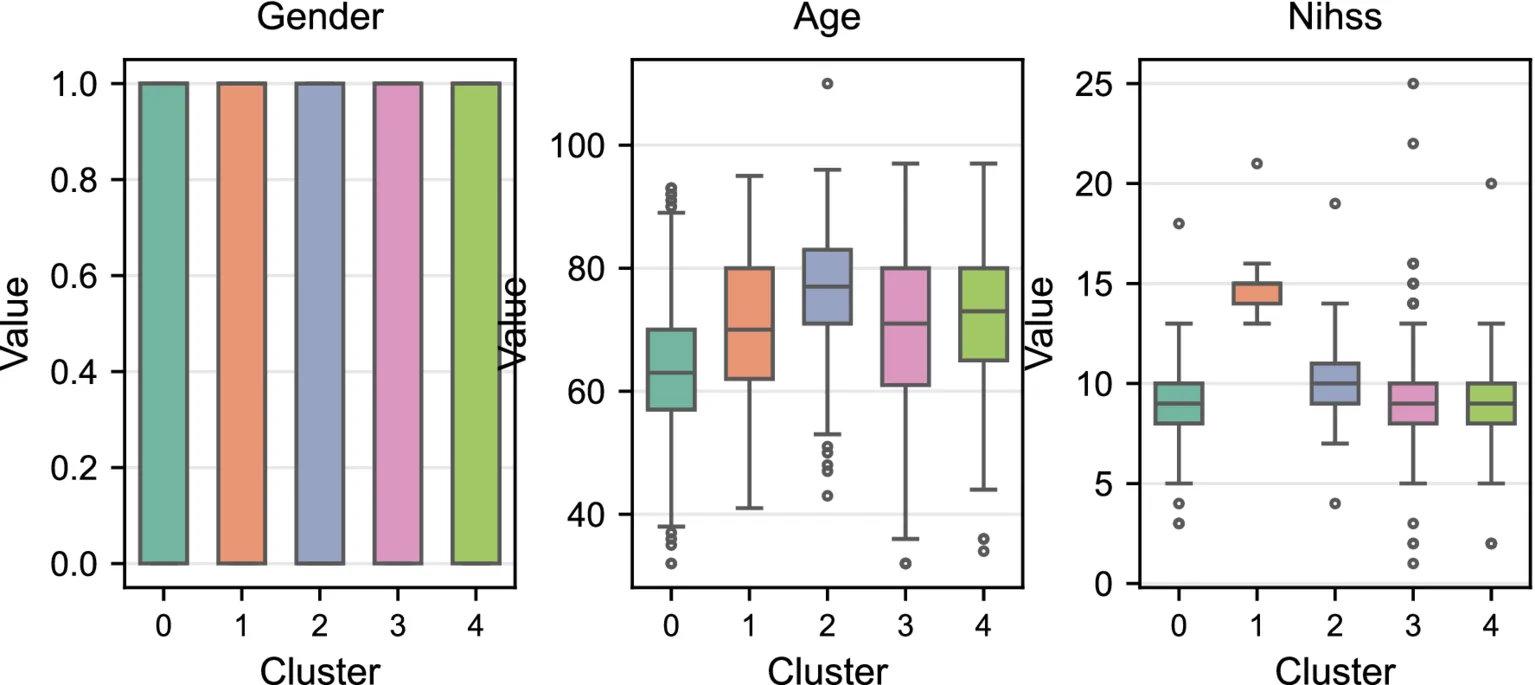
Demographic and clinical characteristics across clusters. This figure displays bar charts or grouped visualizations comparing key demographic and clinical parameters including age, gender distribution, NIHSS score, and other baseline characteristics across the five patient clusters, allowing visual comparison of phenotypic differences between subgroups.

Through cluster analysis, the research subjects were successfully divided into five subgroups (Cluster 0–4) (see Figure 3). This clustering structure enabled in-depth exploration of differences among subgroups in terms of demographic characteristics, clinical indicators, ultrasound plaque characteristics, and mortality. Specifically, the sample sizes were: Cluster 0 (*n* = 1,099, 29.2%), Cluster 1 (*n* = 127, 3.4%), Cluster 2 (*n* = 1,130, 30.0%), Cluster 3 (*n* = 897, 23.8%), and Cluster 4 (*n* = 511, 13.6%). This provides a fundamental framework for further analyzing the health status and disease patterns of each subgroup. As shown in Figure 4, Table 1, these exploratory clusters form a clear structural basis for subsequent comparative analyses. All the statistical results of full and cluster description and comparation is in extended Supplementary Tables 1–12.

**Table 1:** Demographic characteristics and neurological function scores by cluster.

Variable	Cluster_0	Cluster_1	Cluster_2	Cluster_3	Cluster_4
Mortality rate	2.9% (*n* = 32/1099)	8.6% (*n* = 11/127)	8.4 (*n* = 95/1130)	8.6 (*n* = 77/897)	11.2 (*n* = 57/511)
Gender	0.64 ± 0.48	0.57 ± 0.5	0.38 ± 0.49	0.54 ± 0.5	0.53 ± 0.5
	1.0 [0.0–1.0]	1.0 [0.0–1.0]	0.0 [0.0–1.0]	1.0 [0.0–1.0]	1.0 [0.0–1.0]
Age	64.17 ± 10.81	71.16 ± 11.33	77.15 ± 8.16	69.93 ± 12.17	71.95 ± 10.72
	63.0 [57.0–70.0]	70.0 [62.0–80.0]	77.0 [71.0–83.0]	71.0 [61.0–80.0]	73.0 [65.0–80.0]
NIHSS	8.74 ± 1.29	14.81 ± 0.91	9.86 ± 1.21	8.95 ± 2.06	9.18 ± 1.53
	9.0 [8.0–10.0]	15.0 [14.0–15.0]	10.0 [9.0–11.0]	9.0 [8.0–10.0]	9.0 [8.0–10.0]

Data are presented as mean ± standard deviation and median [interquartile range]. The table summarizes baseline patient demographics and acute stroke severity across the five clusters: units: age (years); NIHSS (points); mortality rate (*n*, %). Gender reported as male = 1.

It should be noted that Cluster 1 contained the fewest subjects, accounting for only a small proportion of the overall cohort. The limited sample size in this subgroup may restrict the representativeness and robustness of findings specific to Cluster 1. In particular, extreme values or apparent “outlier” patterns in this cluster may be more susceptible to sampling variability, and thus should be interpreted with caution rather than assumed to reflect the broader patient population.

### Analysis of demographic and neurological function scores

There were marked differences in demographic characteristics and neurological function scores among the five clusters. With respect to age, Cluster 2 comprised the oldest patients, whereas Cluster 0 included the youngest. Clusters 1 and 4 occupied an intermediate age range, and Cluster 3 showed the greatest dispersion in age. These differences suggest that each cluster may represent a distinct combination of age-related physiological status, risk factor profiles, and disease susceptibility. The distributions of age across clusters are illustrated in Figure 4 and summarized in Table 1.

Neurological deficit, assessed by the National Institutes of Health Stroke Scale (NIHSS), also varied substantially across clusters. Cluster 1 exhibited the most severe neurological impairment, with clearly higher NIHSS scores than all other clusters, whereas Cluster 0 had the mildest neurological deficits. The remaining clusters displayed intermediate levels of impairment. However, given the small sample size of Cluster 1, the extremely high NIHSS scores observed in this subgroup may overestimate the prevalence of profoundly severe stroke phenotypes in the general population and may partly reflect sampling fluctuation rather than a stable, distinct clinical class. The between-cluster differences in NIHSS are visualized in Figure 4 and detailed in Table 1.

Sex distribution fluctuated considerably across clusters. Cluster 0 showed the highest proportion of males, whereas Cluster 2 had the lowest. Clusters 3 and 4 had similar male predominance, and Cluster 1 was close to the overall cohort pattern. These differences may reflect sex-related variation in disease occurrence, presentation, or referral patterns, but could also be influenced by sampling. The gender ratios for each cluster are presented in Figure 4, Table 1.

To avoid implying normality for skewed variables, the detailed distributions of age and NIHSS within each cluster are summarized in Table 1 using appropriate descriptive measures (mean ± SD and/or median [IQR] according to the underlying distribution), and between-cluster comparisons were performed using parametric or non-parametric tests as appropriate.

### Analysis of inflammatory and blood indicators

**Figure 5:**
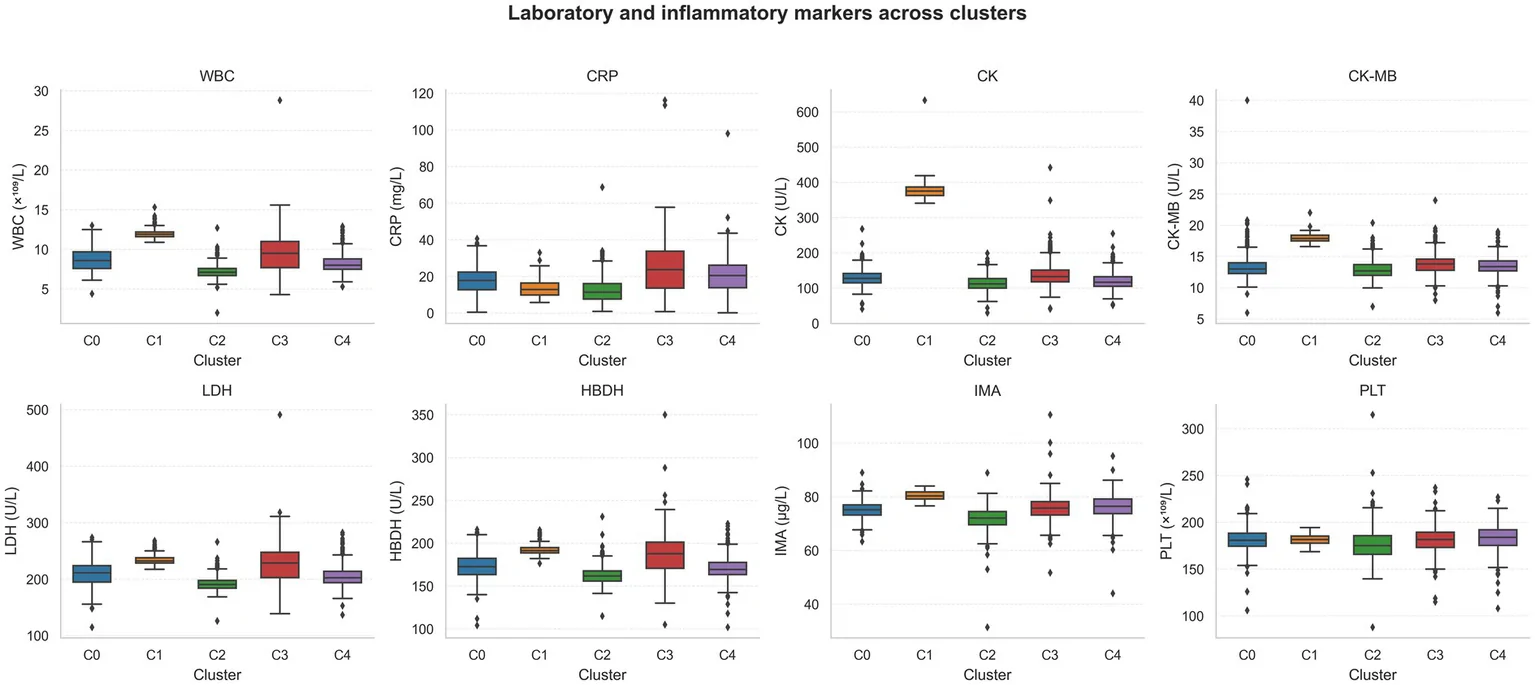
Laboratory and inflammatory markers across clusters. This figure presents comprehensive visualization of inflammatory and laboratory markers such as WBC, CRP, and CK across clusters using multiple subplots. The inter-cluster variations in these acute phase reactants and inflammatory biomarkers are clearly displayed.

Inflammatory and hematological markers showed clear heterogeneity across clusters. Cluster 1 may represent a preliminary exploratory phenotype characterized by extreme values, but these findings should be interpreted cautiously because of the small sample size. This pattern is consistent with the severe neurological deficits observed in Cluster 1 and suggests that this cluster may represent patients with highly active systemic inflammation and multi-organ stress. Nevertheless, because Cluster 1 contains relatively few patients, these extreme elevations could represent a non-typical subset and should not be generalized uncritically. The distributions of these inflammatory markers across clusters are depicted in Figure 5 and summarized in Table 2.

**Table 2:** Inflammatory and hematological indicators by cluster.

Variable	Cluster_0	Cluster_1	Cluster_2	Cluster_3	Cluster_4
WBC	8.7 ± 1.34	12.0 ± 0.73	7.17 ± 0.75	9.59 ± 2.21	8.27 ± 1.17
	8.6 [7.6–9.7]	11.9 [11.6–12.2]	7.1 [6.7–7.6]	9.5 [7.8–11.0]	8.0 [7.5–8.8]
CRP	17.75 ± 6.66	13.99 ± 5.04	12.54 ± 6.24	24.41 ± 12.86	20.37 ± 9.26
	17.9 [12.7–22.4]	12.9 [9.9–16.5]	11.4 [7.7–16.08]	23.9 [13.9–33.9]	20.5 [13.9–26.2]
CK	129.7 ± 20.7	376.53 ± 27.36	114.26 ± 20.45	137.24 ± 30.69	120.06 ± 23.55
	127.7 [115.05–141.45]	375.0 [362.8–385.7]	112.55 [100.4–127.4]	133.2 [117.9–151.4]	116.7 [105.0–132.35]
LDH	210.66 ± 19.96	234.8 ± 9.89	191.64 ± 10.58	228.01 ± 30.68	205.27 ± 18.46
	211.5 [195.0–224.1]	232.0 [228.45–238.0]	190.6 [184.0–197.9]	228.9 [203.0–247.8]	202.5 [193.85–214.1]
HBDH	172.97 ± 14.05	192.47 ± 7.02	162.24 ± 9.17	187.36 ± 20.63	170.96 ± 13.51
	172.7 [163.4–182.3]	191.2 [188.65–194.85]	161.5 [155.72–167.58]	188.0 [170.9–201.1]	169.5 [163.3–178.0]
CK-MB	13.28 ± 1.66	17.92 ± 0.7	12.9 ± 1.32	13.8 ± 1.52	13.46 ± 1.5
	13.0 [12.3–14.0]	17.9 [17.45–18.4]	12.7 [12.0–13.7]	13.8 [12.8–14.6]	13.4 [12.7–14.3]
IMA	75.03 ± 2.8	80.45 ± 1.85	71.97 ± 3.71	75.6 ± 3.89	76.24 ± 4.35
	75.2 [73.3–77.0]	80.3 [79.2–81.7]	72.0 [69.6–74.5]	75.8 [73.2–78.2]	76.5 [73.8–79.2]
PLT	181.62 ± 11.01	181.97 ± 5.22	176.27 ± 15.03	181.01 ± 12.46	183.09 ± 13.25
	181.0 [174.6–188.5]	181.7 [177.95–185.5]	175.2 [165.8–185.9]	181.6 [173.3–189.4]	184.0 [175.35–192.05]

This table compares white blood cell counts, inflammatory markers (CRP), muscle enzymes (CK, CK-MB), and other hematological parameters to assess systemic inflammation and tissue injury levels. Units: WBC (×10^9^/L); CRP (mg/L); CK, CK-MB, LDH, HBDH (U/L); IMA (μg/L); PLT (×10^9^/L).

Data are presented as mean ± standard deviation and median [interquartile range].

Cluster 3, in contrast, exhibited the highest C-reactive protein (CRP) levels among all clusters, pointing to a persistent inflammatory state that is not necessarily accompanied by the same degree of acute muscle enzyme elevation seen in Cluster 1. This may indicate a more chronic or smoldering inflammatory phenotype in Cluster 3. Other hematological indices, such as red blood cell count and platelet count, also showed statistically detectable but clinically modest variations between clusters. These differences could influence blood rheology and microcirculatory function and may in turn affect disease progression and prognosis. The full set of inflammatory and hematological indicators is presented in Figure 5, Table 2.

Given that many inflammatory markers (e.g., WBC, CRP, CK) showed skewed distributions, they are summarized in Table 2 using median [IQR] or mean ± SD, as appropriate to their distributional properties, and non-parametric tests were employed for between-cluster comparisons where normality was not met.

### Analysis of biochemical and metabolic indicators

**Figure 6:**
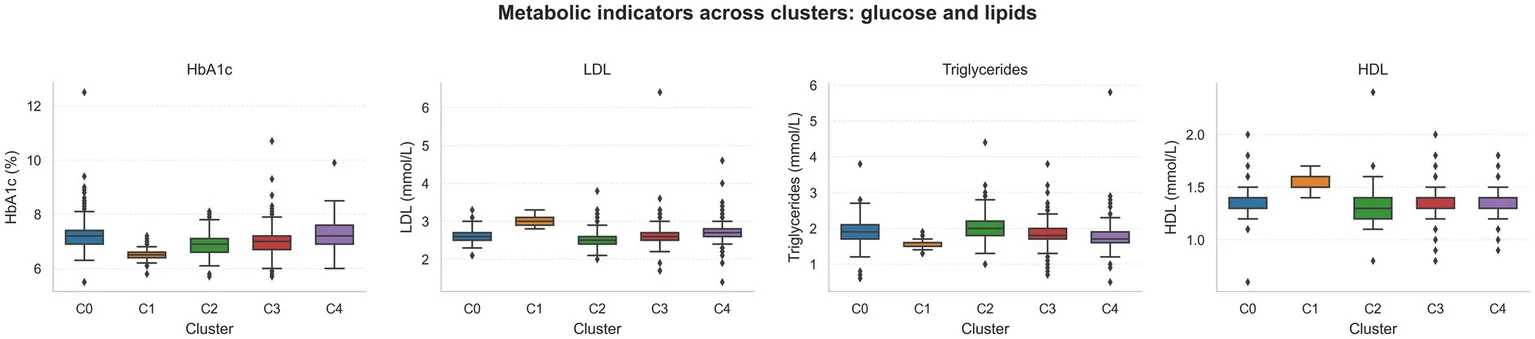
Metabolic indicators part 1 across clusters. This figure displays box-and-whisker plots or similar visualizations of metabolic parameters including HbA1c, LDL cholesterol, triglycerides, and HDL cholesterol across five clusters, showing differences in glucose and lipid metabolism among patient subgroups.

**Figure 7:**
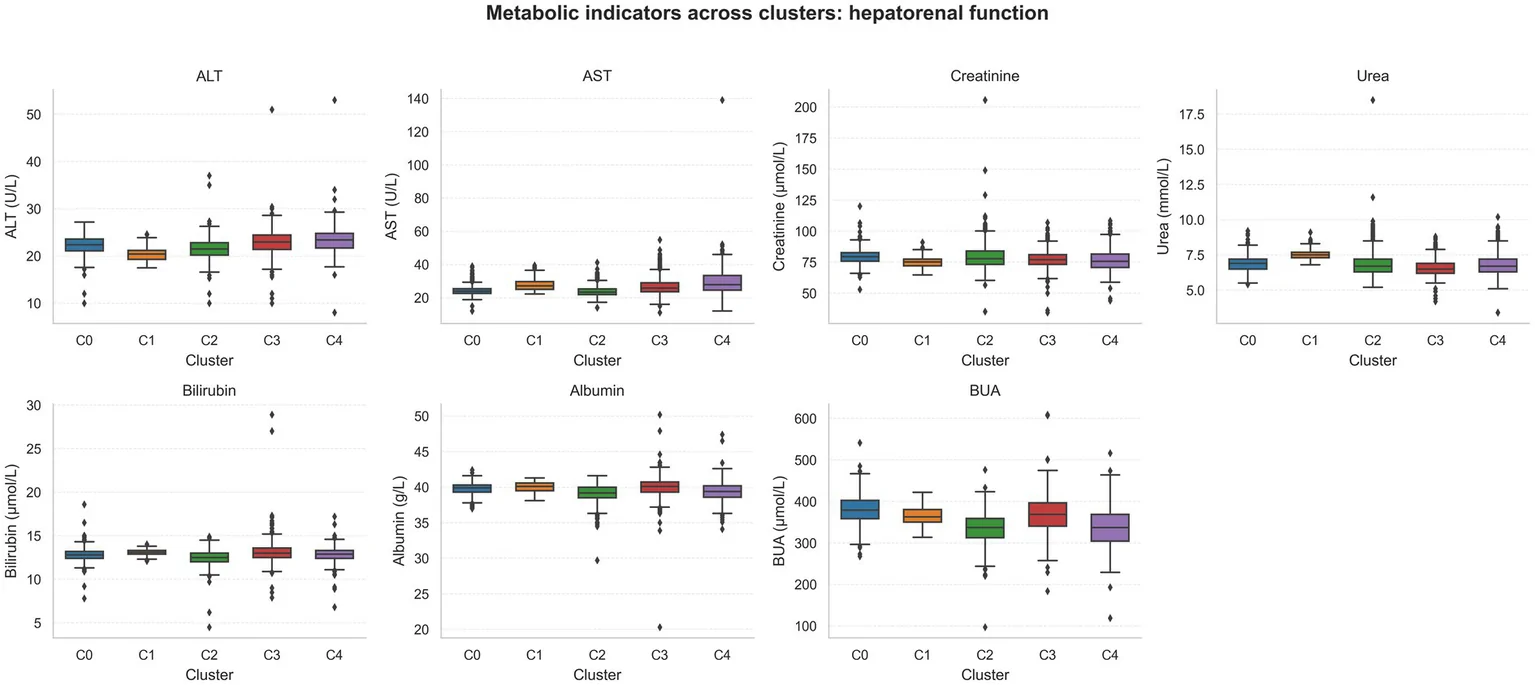
Metabolic indicators part 2 across clusters. This figure presents additional metabolic and hepatorenal function markers including ALT, AST, creatinine, and urea across clusters, visualizing liver and kidney function status and metabolic parameters in different patient subgroups.

Distinct differences in biochemical and metabolic indicators were observed across clusters. Clusters 0 and 4 were particularly notable. For liver enzyme alanine aminotransferase (ALT), Cluster 0 tended to have lower values, suggesting relatively mild hepatocellular injury, whereas Cluster 4 tended to show higher ALT levels, consistent with more pronounced hepatic stress or metabolic disturbance. These patterns are reflected in the distributions shown in Figures 6, 7 and the summary statistics in Tables 3, 4.

**Table 3:** Biochemical indicators (glucose and lipid metabolism) by cluster.

Variable	Cluster_0	Cluster_1	Cluster_2	Cluster_3	Cluster_4
HbA1c	7.2 ± 0.44	6.53 ± 0.23	6.92 ± 0.36	6.99 ± 0.39	7.22 ± 0.45
	7.2 [6.9–7.4]	6.5 [6.4–6.65]	6.9 [6.6–7.2]	7.0 [6.7–7.2]	7.2 [6.9–7.6]
LDL	2.63 ± 0.15	3.0 ± 0.11	2.55 ± 0.18	2.62 ± 0.23	2.7 ± 0.25
	2.6 [2.5–2.7]	3.0 [2.9–3.1]	2.5 [2.4–2.6]	2.6 [2.5–2.7]	2.7 [2.6–2.8]
TG	1.9 ± 0.26	1.56 ± 0.11	2.01 ± 0.32	1.85 ± 0.27	1.77 ± 0.31
	1.9 [1.7–2.1]	1.6 [1.5–1.6]	2.0 [1.8–2.2]	1.8 [1.7–2.0]	1.7 [1.6–1.9]
HDL	1.35 ± 0.1	1.52 ± 0.06	1.31 ± 0.11	1.36 ± 0.12	1.36 ± 0.11
	1.3 [1.3–1.4]	1.5 [1.5–1.55]	1.3 [1.2–1.4]	1.4 [1.3–1.4]	1.4 [1.3–1.4]

The table presents glycemic control (HbA1c) and lipid profile (LDL, HDL, TG) data to evaluate metabolic risk factors among the clusters. Units: HbA1c (%); LDL, HDL (mmol/L); TG (mmol/L).

Data are presented as mean ± standard deviation and median [interquartile range].

**Table 4:** Biochemical indicators (hepatorenal function) by cluster. Data are presented as mean ± standard deviation and median [interquartile range].

Variable	Cluster_0	Cluster_1	Cluster_2	Cluster_3	Cluster_4
ALT	22.35 ± 1.83	20.34 ± 1.49	21.52 ± 1.99	22.89 ± 2.5	23.35 ± 2.82
	22.4 [21.1–23.6]	20.3 [19.25–21.2]	21.5 [20.2–22.8]	23.0 [21.5–24.5]	23.4 [21.7–24.8]
AST	24.23 ± 2.55	27.79 ± 3.73	23.87 ± 3.03	26.81 ± 4.73	29.73 ± 8.24
	24.0 [22.6–25.4]	27.0 [25.05–29.7]	23.4 [21.9–25.2]	25.8 [23.6–29.1]	27.9 [24.6–33.35]
Creatinine	79.35 ± 5.53	75.07 ± 4.76	79.24 ± 9.3	77.15 ± 6.62	76.69 ± 8.48
	79.5 [75.95–82.7]	75.1 [71.95–77.75]	77.8 [73.12–84.0]	77.0 [73.2–81.0]	75.6 [70.8–81.35]
Urea	6.87 ± 0.52	7.54 ± 0.42	6.83 ± 0.79	6.61 ± 0.56	6.81 ± 0.77
	6.9 [6.5–7.2]	7.5 [7.3–7.7]	6.7 [6.3–7.2]	6.5 [6.2–6.9]	6.7 [6.3–7.2]
Bilirubin	12.81 ± 0.72	13.09 ± 0.37	12.46 ± 0.82	13.11 ± 1.16	12.82 ± 0.87
	12.8 [12.4–13.2]	13.1 [12.9–13.3]	12.5 [11.9–13.0]	13.0 [12.5–13.6]	12.9 [12.4–13.3]
Albumin	39.76 ± 0.76	40.06 ± 0.65	39.12 ± 1.16	40.05 ± 1.4	39.37 ± 1.31
	39.9 [39.3–40.3]	40.1 [39.5–40.6]	39.2 [38.5–40.0]	40.1 [39.3–40.8]	39.4 [38.6–40.2]
BUA	380.58 ± 34.21	365.79 ± 22.73	335.26 ± 33.92	368.94 ± 43.47	337.2 ± 44.08
	379.0 [358.4–402.3]	362.1 [349.75–380.25]	337.1 [312.6–359.18]	368.7 [341.6–396.5]	336.8 [304.8–368.85]

This table summarizes liver enzyme levels (ALT, AST), kidney function markers (Creatinine, Urea, BUA), and protein levels (Albumin, Bilirubin) to assess organ function across clusters. Units: ALT, AST (U/L); Creatinine (μmol/L); Urea (mmol/L); Bilirubin (μmol/L); Albumin (g/L); BUA (μmol/L).

Regarding kidney function, serum creatinine values in Cluster 0 lay toward the lower end of the normal range, indicating relatively preserved glomerular filtration. In contrast, patients in Cluster 4 exhibited creatinine values shifted toward higher levels, suggesting early or mild renal impairment. Although absolute differences were not large, the systematic shift in distribution is consistent with a higher burden of organ dysfunction in Cluster 4. These kidney function differences are summarized in Figures 6, 7 and Tables 3, 4.

Among Clusters 1, 2, and 3, distinct patterns were also evident, particularly for aspartate aminotransferase (AST). Cluster 1 showed comparatively high AST levels, in line with its severe inflammatory and tissue injury profile. Cluster 2 maintained comparatively lower AST values, consistent with milder hepatic and systemic injury and a more stable physiological state. Cluster 3 lay between these extremes, suggesting an intermediate degree of inflammation-related hepatic and tissue impact. These biochemical and metabolic differences may partly explain the variation in clinical outcomes and could inform more individualized management strategies. The cluster-specific distributions of AST, ALT, and related metabolic indicators are displayed in Figures 6, 7 and detailed in Tables 3, 4.

Because many biochemical markers (e.g., AST, ALT, creatinine) exhibited non-normal distributions in formal testing, their descriptive statistics in Tables 3, 4 are presented using median [IQR] or mean ± SD depending on distribution, and appropriate non-parametric tests were used when normality assumptions were violated.

### Analysis of coagulation indicators and blood gas indicators

**Figure 8:**
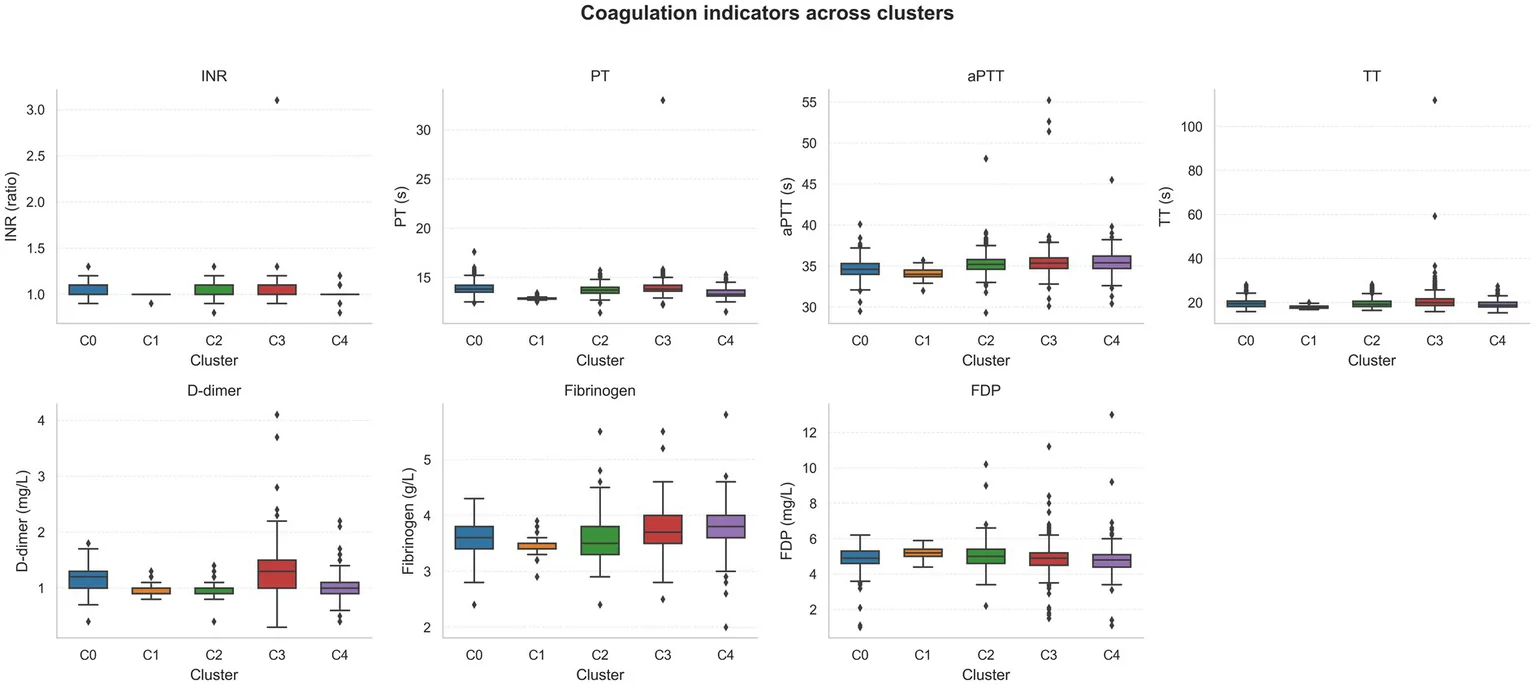
Coagulation indicators across clusters. This figure displays coagulation parameters including INR, PT, aPTT, D-dimer, and fibrinogen across five clusters, illustrating inter-cluster variations in thrombotic tendency and coagulation function.

**Figure 9:**
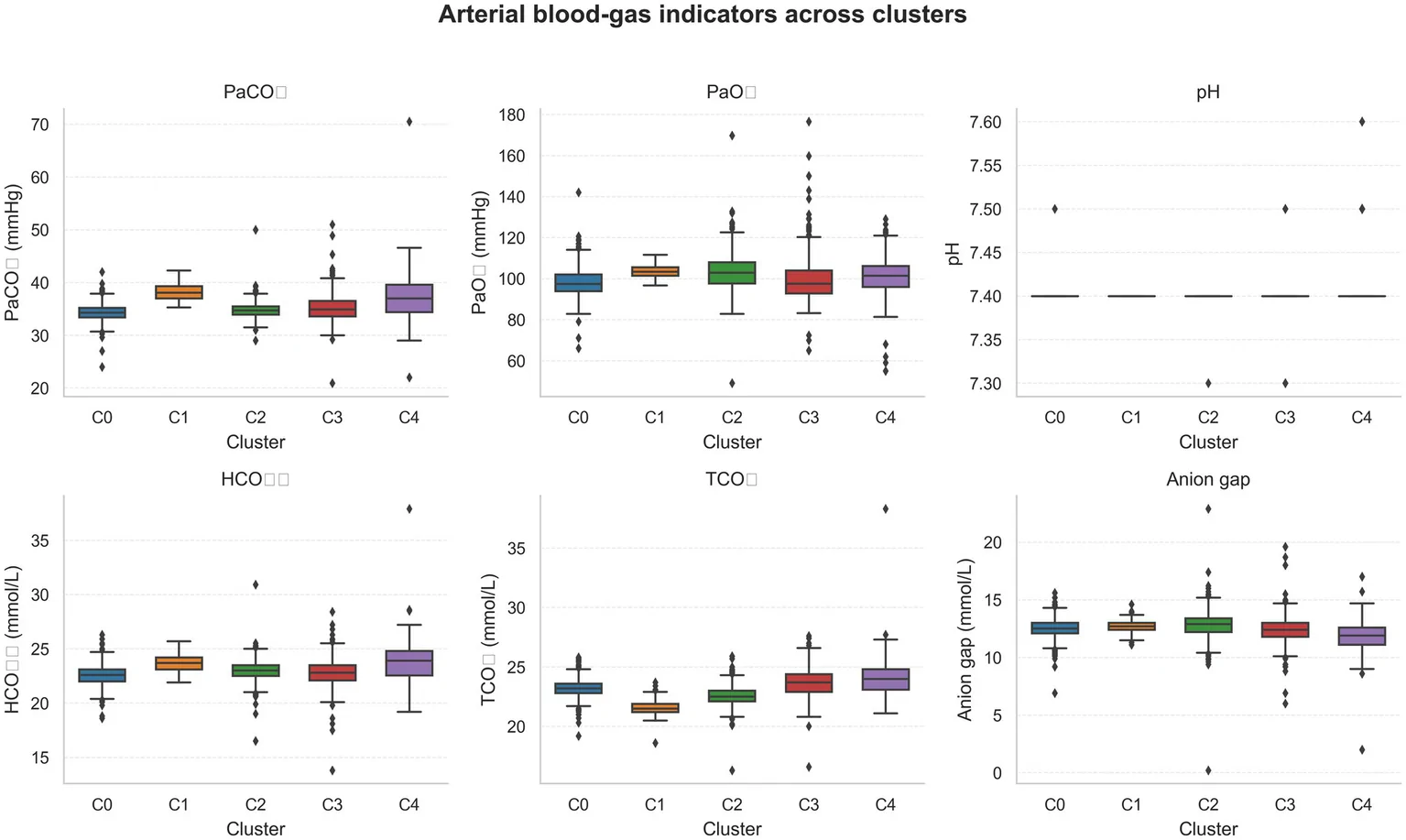
Blood gas indicators across clusters. This figure presents arterial blood gas parameters including PaCO_2_, PaO_2_, pH, and HCO_3_^−^ across clusters, reflecting respiratory and metabolic acid–base status differences among patient subgroups.

Coagulation parameters and blood gas indices further differentiated the clusters. For coagulation, prothrombin time (PT) in Clusters 0 and 4 was generally longer than in Cluster 1, whereas Cluster 1 showed the shortest PT values with very limited variability. D-dimer concentrations were particularly elevated in Cluster 3 compared with other clusters, highlighting enhanced fibrin turnover and potential thrombotic activity in this subgroup. Fibrinogen levels were highest in Cluster 4 and lowest in Cluster 1, suggesting differences in fibrinogen synthesis, consumption, or both. Together, these findings indicate that Clusters 3 and 4 may be characterized by a more pro-thrombotic milieu, whereas Cluster 1 shows a different pattern of coagulation activation. These coagulation profiles are illustrated in Figures 8, 9 and summarized in Tables 5, 6.

At the blood gas level, partial pressure of carbon dioxide (PaCO₂) and total carbon dioxide (tCO₂) displayed notable inter-cluster differences. Cluster 0 had the lowest PaCO₂ values, implying relatively efficient ventilation and carbon dioxide elimination. In contrast, Clusters 1 and 4 exhibited higher PaCO₂ values, consistent with impaired carbon dioxide excretion and possible respiratory insufficiency or reduced ventilatory drive. tCO₂ was also lower in Cluster 1 than in other clusters, indicating distinct patterns of acid–base handling and CO₂ buffering.

With respect to oxygenation, PaO₂ values in Clusters 1 and 2 were similar and relatively high, whereas Cluster 3 tended to have lower and more variable PaO₂, pointing to comparatively weaker oxygenation in this subgroup. Bicarbonate (HCO₃^−^) levels were highest in Cluster 1 and lowest in Cluster 3, suggesting that clusters differ in the degree and direction of metabolic compensation for respiratory or systemic acid–base disturbances. These differences in blood gas indices are clinically relevant for assessing respiratory and metabolic function in each cluster and can suggest potential hypotheses for tailoring ventilatory and metabolic management. The distributions of coagulation and blood gas parameters across clusters are presented in Figures 8, 9, Tables 5, 6.

**Table 5:** Coagulation indicators by cluster. Data are presented as mean ± standard deviation and median [interquartile range].

Variable	Cluster_0	Cluster_1	Cluster_2	Cluster_3	Cluster_4
INR	1.06 ± 0.06	1.0 ± 0.01	1.04 ± 0.05	1.07 ± 0.09	1.02 ± 0.05
	1.1 [1.0–1.1]	1.0 [1.0–1.0]	1.0 [1.0–1.1]	1.1 [1.0–1.1]	1.0 [1.0–1.0]
PT	13.87 ± 0.5	12.85 ± 0.17	13.72 ± 0.41	13.92 ± 0.82	13.41 ± 0.45
	13.8 [13.5–14.2]	12.8 [12.8–12.9]	13.7 [13.4–14.0]	13.8 [13.6–14.2]	13.3 [13.1–13.7]
aPTT	34.65 ± 0.94	34.06 ± 0.6	35.22 ± 1.07	35.42 ± 1.29	35.47 ± 1.21
	34.6 [34.0–35.3]	34.0 [33.65–34.5]	35.2 [34.6–35.8]	35.3 [34.7–36.0]	35.4 [34.7–36.2]
D-dimer	1.16 ± 0.21	0.95 ± 0.12	0.96 ± 0.1	1.3 ± 0.36	1.01 ± 0.2
	1.2 [1.0–1.3]	0.9 [0.9–1.0]	1.0 [0.9–1.0]	1.3 [1.0–1.5]	1.0 [0.9–1.1]
Fibrinogen	3.6 ± 0.23	3.44 ± 0.15	3.55 ± 0.31	3.72 ± 0.34	3.81 ± 0.37
	3.6 [3.4–3.8]	3.4 [3.4–3.5]	3.5 [3.3–3.8]	3.7 [3.5–4.0]	3.8 [3.6–4.0]
FDP	4.9 ± 0.55	5.18 ± 0.32	5.02 ± 0.62	4.84 ± 0.63	4.81 ± 0.72
	4.9 [4.6–5.3]	5.2 [5.0–5.4]	5.0 [4.6–5.4]	4.9 [4.5–5.2]	4.8 [4.4–5.1]

The table displays coagulation times (PT, aPTT), INR, fibrinogen, and thrombotic markers (D-dimer, FDP) to characterize hemostatic status. Units: INR (ratio); PT (s); aPTT (s); D-dimer (mg/L); Fibrinogen (g/L); FDP (mg/L).

**Table 6:** Arterial blood gas indicators by cluster. Data are presented as mean ± standard deviation and median [interquartile range].

Variable	Cluster_0	Cluster_1	Cluster_2	Cluster_3	Cluster_4
PaCO_2_	34.31 ± 1.45	38.21 ± 1.56	34.78 ± 1.38	35.22 ± 2.22	37.12 ± 3.61
	34.3 [33.4–35.2]	38.1 [36.95–39.35]	34.6 [33.9–35.5]	34.9 [33.7–36.5]	37.0 [34.4–39.6]
PaO_2_	97.95 ± 6.32	103.64 ± 3.25	102.94 ± 7.89	98.92 ± 9.3	101.01 ± 8.49
	97.4 [93.9–102.0]	103.3 [101.4–105.65]	102.85 [97.52–107.9]	97.4 [92.7–103.6]	101.2 [95.8–106.1]
pH	7.4 ± 0.0	7.4 ± 0.0	7.4 ± 0.0	7.4 ± 0.01	7.41 ± 0.03
	7.4 [7.4–7.4]	7.4 [7.4–7.4]	7.4 [7.4–7.4]	7.4 [7.4–7.4]	7.4 [7.4–7.4]
HCO_3_	22.56 ± 0.84	23.63 ± 0.78	23.0 ± 0.88	22.82 ± 1.08	23.79 ± 1.63
	22.6 [22.0–23.1]	23.7 [23.1–24.15]	23.0 [22.5–23.5]	22.8 [22.1–23.5]	23.9 [22.5–24.8]

This table summarizes arterial blood gas parameters including pH, partial pressures of oxygen and carbon dioxide, and bicarbonate levels to evaluate respiratory and metabolic status. Units: PaCO₂, PaO₂ (mmHg); pH (units); HCO_3_^−^ (mmol/L).

Again, because key blood gas and coagulation variables showed deviations from normality, descriptive statistics in Tables 5, 6 use median [IQR] for skewed variables, and non-parametric tests were employed where appropriate.

### Analysis of ultrasound plaque characteristics

**Figure 10:**
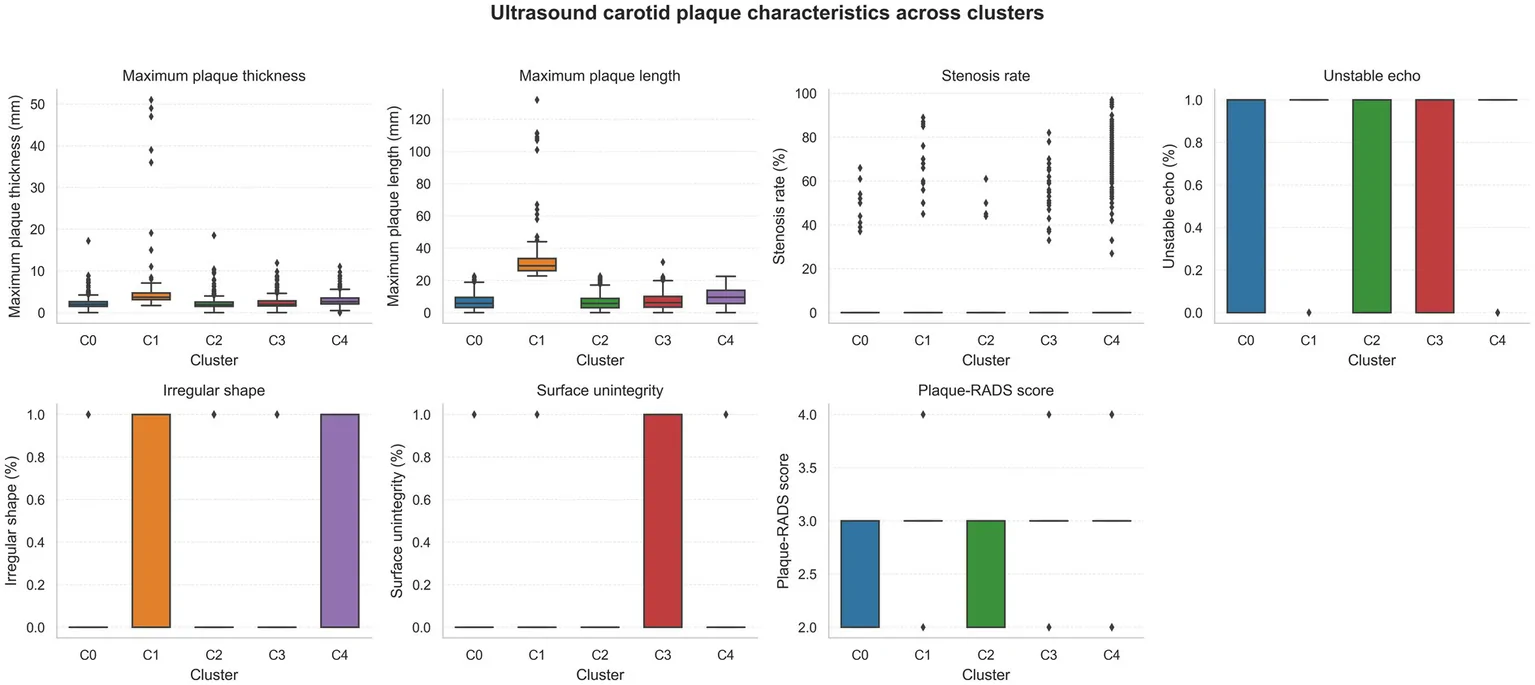
Ultrasound plaque characteristics across clusters. This figure comprehensively displays carotid plaque characteristics including maximum thickness, length, stenosis rate, echogenicity pattern, surface regularity, surface integrity, and Plaque-RADS score across five clusters, showing substantial inter-cluster variations in plaque morphology and stability features.

Ultrasound plaque characteristics revealed striking differences among clusters. Cluster 1 showed the greatest maximum plaque thickness and length, together with the highest proportion of unstable echo patterns, indicating a large plaque burden with extremely poor stability. Such plaques are at high risk for rupture and thrombus formation, which can critically impair arterial patency and downstream perfusion. However, given the small number of patients in Cluster 1, these extreme plaque features may over-represent the true distribution in the broader population and might partly result from sampling variation. These patterns are clearly illustrated in Figure 10 and summarized in Table 7.

**Table 7:** Ultrasound carotid plaque characteristics by cluster. Data are presented as mean ± standard deviation and median [interquartile range].

Variable	Cluster_0	Cluster_1	Cluster_2	Cluster_3	Cluster_4
Max thickness	2.14 ± 1.18	5.61 ± 8.21	2.03 ± 1.16	2.26 ± 1.21	2.84 ± 1.28
	2.0 [1.5–2.6]	3.7 [3.05–4.7]	1.9 [1.5–2.5]	2.0 [1.6–2.8]	2.7 [2.1–3.5]
Max length	6.55 ± 4.82	34.43 ± 19.15	6.23 ± 4.68	6.97 ± 5.11	9.79 ± 5.72
	5.8 [3.15–9.5]	29.0 [25.85–33.4]	5.65 [3.0–8.78]	6.1 [3.4–10.0]	9.5 [5.75–13.8]
Stenosis rate	0.4 ± 4.53	8.56 ± 23.15	0.18 ± 3.0	1.88 ± 10.32	15.84 ± 29.94
	0.0 [0.0–0.0]	0.0 [0.0–0.0]	0.0 [0.0–0.0]	0.0 [0.0–0.0]	0.0 [0.0–0.0]
Unstable echo	0.55 ± 0.5	0.93 ± 0.26	0.48 ± 0.5	0.59 ± 0.49	0.78 ± 0.41
	1.0 [0.0–1.0]	1.0 [1.0–1.0]	0.0 [0.0–1.0]	1.0 [0.0–1.0]	1.0 [1.0–1.0]
Irregular shape	0.19 ± 0.39	0.36 ± 0.48	0.13 ± 0.33	0.2 ± 0.4	0.36 ± 0.48
	0.0 [0.0–0.0]	0.0 [0.0–1.0]	0.0 [0.0–0.0]	0.0 [0.0–0.0]	0.0 [0.0–1.0]
Surface unintegrity	0.01 ± 0.08	0.09 ± 0.29	0.0 ± 0.0	0.46 ± 0.5	0.04 ± 0.18
	0.0 [0.0–0.0]	0.0 [0.0–0.0]	0.0[0.0–0.0]	0.0 [0.0–1.0]	0.0 [0.0–0.0]
RADS score	2.64 ± 0.48	3.02 ± 0.32	2.56 ± 0.5	2.8 ± 0.41	2.95 ± 0.53
	3.0 [2.0–3.0]	3.0 [3.0–3.0]	3.0 [2.0–3.0]	3.0 [3.0–3.0]	3.0 [3.0–3.0]

The table provides detailed ultrasound measurements of carotid plaques, including dimensions (thickness, length), morphology (shape, surface integrity), echogenicity, and standardized risk scores (Plaque-RADS). Units: max thickness, max length (mm); stenosis rate (%); unstable echo, irregular shape, surface unintegrity (*n*, proportion); plaque-RADS (score).

Cluster 4 exhibited the highest stenosis rate, reflecting a substantial risk of vascular occlusion, which may lead to insufficient blood supply to the corresponding organs and serious impairment of organ function. Clusters 0 and 2 had minimal stenosis, suggesting a relatively low structural burden of carotid disease despite clinical stroke. Cluster 3 showed intermediate stenosis and a high frequency of surface integrity disruption (plaque ulceration), pointing to a distinct pattern of vulnerable plaque morphology.

Differences in plaque morphology (e.g., thickness, length, irregular shape), stability (unstable echo, ulceration), and composite risk scoring across clusters are of great importance for predicting cardiovascular and cerebrovascular event risk and for designing personalized treatment strategies. The observed differences in plaque morphology, stability and stenosis degree across clusters highlight the heterogeneity of carotid disease burden among patients. Clusters with more advanced plaque features may represent higher-risk phenotypes that require closer clinical attention and more rigorous risk factor control. The distribution of ultrasound plaque characteristics for each cluster is summarized in Figure 10, Table 7.

### Mortality analysis

The differences in 30-day mortality rates among different clusters are statistically significant. Cluster 4 has the highest 30-day mortality rate, which is significantly higher than that of Cluster 0. Combining with the characteristics of various indicators mentioned above, the relatively high mortality rate of Cluster 4 may be related to its high vascular stenosis rate and abnormal related metabolic indicators, suggesting a poor prognosis for patients in this subgroup. The mortality rates of Cluster 1, Cluster 2, and Cluster 3 are relatively close and are all higher than that of Cluster 0, but the differences compared with Cluster 4 do not reach statistical significance. Given the small sample size of Cluster 1, its mortality rate might not be a reliable reflection of a consistent subgroup mortality in the overall patient population. The small number of subjects in this cluster could introduce a higher degree of uncertainty in the estimated mortality figure.

### Univariable and multivariable regression analysis

After statistically analysis of mortality, univariable and multivariate logistic regression analysis was conducted to determine independent predictors of 30-day mortality in Tables 8, 9. Multiple variables were identified as significant influencing factors *p* < 0.05. Risk factors for mortality included age (OR = 1.0272, 95%CI: 1.0076–1.0471), CK_MB (OR = 1.4568, 95%CI: 1.3029–1.6290), TT (OR = 1.1402, 95%CI: 1.0586–1.2281), serum potassium (K, OR = 49.2965, 95%CI: 8.2336–295.1494), calcium (Ca, OR = 73174140668.3516, 95%CI: 412323120.4724–12986065046308.754), phosphorus (P, OR = 475.0334, 95%CI: 12.3760–18233.3715) and PaO_2_ (OR = 1.1469, 95%CI: 1.1180–1.1766). In contrast, FDP (OR = 0.4135, 95%CI: 0.3130–0.5462), plasma viscosity (OR = 0.0106, 95%CI: 0.0001–0.7962) and bilirubin (OR = 0.2440, 95%CI: 0.1880–0.3166) served as protective factors. With Cluster 0 as the reference group, Cluster 2 (OR = 2.2957, 95%CI: 1.3376–3.9402, *p* = 0.0026), Cluster 3 (OR = 3.3327, 95%CI: 1.9469–5.7050, *p* < 0.001) and Cluster 4 (OR = 4.2487, 95%CI: 2.4006–7.5197, *p* < 0.001) were independently associated with increased mortality risk. Moreover, INR, max_length and pH showed marginally significant associations with 30-day mortality (0.05 ≤ *p* < 0.20).

**Table 8:** Univariable logistic regression analysis of baseline variables associated with 30-day mortality in ischemic stroke patients with carotid plaques.

Variable	Coefficient	OR	95% CI	*p* value
Gender	0.16	1.17	0.92–1.50	0.208
Age	0.11	1.11	1.10–1.13	<0.001
NIHSS	0.25	1.28	1.21–1.35	<0.001
WBC	−0.07	0.93	0.87–1.00	0.055
RBC	−4.33	0.01	0.01–0.03	<0.001
PLT	0.01	1.01	1.00–1.02	0.007
CRP	−0.02	0.98	0.97–1.00	0.026
LDH	0.01	1.01	1.01–1.02	<0.001
HBDH	0.03	1.03	1.02–1.04	<0.001
CK_MB	0.33	1.39	1.30–1.48	<0.001
IMA	−0.09	0.92	0.89–0.94	<0.001
aPTT	1.22	3.38	2.95–3.86	<0.001
TT	0.09	1.09	1.04–1.14	<0.001
Fibrinogen	0.97	2.64	1.82–3.82	<0.001
FDP	−0.44	0.65	0.53–0.79	<0.001
HbA1c	−1.73	0.18	0.13–0.25	<0.001
TG	−1.15	0.32	0.20–0.49	<0.001
LDL	−0.66	0.52	0.28–0.97	0.039
Lactate	−0.82	0.44	0.27–0.70	<0.001
NA	−0.28	0.76	0.68–0.84	<0.001
K	7.51	1828.47	466.40–7168.37	<0.001
Cl	0.55	1.73	1.54–1.94	<0.001
Ca	15.00	3266377.57	194019.34–54990510.98	<0.001
P	15.46	5164643.54	461098.68–57847797.54	<0.001
PaO_2_	0.09	1.09	1.08–1.11	<0.001
HCO_3_	−0.67	0.51	0.45–0.58	<0.001
Anion_gap	−1.00	0.37	0.32–0.42	<0.001
ALT	−0.20	0.82	0.78–0.87	<0.001
Creatinine	0.20	1.23	1.20–1.25	<0.001
Urea	2.57	13.12	10.22–16.83	<0.001
Bilirubin	−1.23	0.29	0.25–0.35	<0.001
Albumin	−0.37	0.69	0.63–0.76	<0.001
Cluster_1	0.19	1.21	0.64–2.27	0.554
Cluster_2	0.24	1.27	0.98–1.65	0.068
Cluster_3	0.25	1.29	0.98–1.69	0.071
Cluster_4	0.58	1.79	1.32–2.43	<0.001

**Table 9:** Multivariable logistic regression analysis of independent predictors of 30-day mortality in ischemic stroke patients with carotid plaques.

Variable	Coefficient	OR	95% CI	*p* value
Age	0.03	1.03	1.01–1.05	0.006
CK_MB	0.38	1.46	1.30–1.63	<0.001
INR	3.51	33.40	0.96–1165.50	0.053
TT	0.13	1.14	1.06–1.23	<0.001
FDP	−0.88	0.41	0.31–0.55	<0.001
Plasma_viscosity	−4.55	0.01	0.00–0.80	0.039
K	3.90	49.30	8.23–295.15	<0.001
Ca	25.02	73174140668.35	412323120.47–12986065046308.75	<0.001
P	6.16	475.03	12.38–18233.37	<0.001
PaO_2_	0.14	1.15	1.12–1.18	<0.001
pH	−10.53	0.00	0.00–264.55	0.200
Bilirubin	−1.41	0.24	0.19–0.32	<0.001
Max_length	−0.02	0.98	0.97–1.00	0.058
Cluster_2	0.83	2.30	1.34–3.94	0.003
Cluster_3	1.20	3.33	1.95–5.71	<0.001
Cluster_4	1.45	4.25	2.40–7.52	<0.001

These findings suggest that prognostic differences persisted after adjustment for measured covariates; however, cluster membership should not be interpreted as proving biological heterogeneity beyond all baseline clinical differences.

## Discussion

### Algorithm-related: advantages of *k*-means and UMAP algorithms in clustering grouping

This study applied *k*-means clustering combined with UMAP dimensionality reduction technology to stratify stroke patients with carotid plaques [commonly classification to TOAST type of LAA (3)] into data-driven subgroups, showing significant advantages over traditional preliminary phenotypic stratification of short-term risk methods and other machine learning algorithms.

Firstly, *k*-means algorithm identifies potential subgroups from multi-dimensional variables from demographics, metabolic indicators to ultrasonic features using unsupervised learning, reducing reliance on single-indicator stratification. Compare with the traditional Plaque-RADS system which integrates plaque morphology and composition and only relies on predefined risk features (17), *k*-means can capture the complex interaction between metabolic and plaque features through data-driven methods which is consistent with the conclusion of Fang et al. (14) who used machine learning to predict plaque vulnerability in gout patients and show that multi-dimensional integrated analysis is superior to a single indicator.

Secondly, UMAP was used for visualization to preserve local structure when projecting high-dimensional data into 2D space, facilitating interpretation of the separation among the five subgroups. Similar visualization benefits have been reported in the study by Omarov et al. (18), which revealed genetically driven subtypes of carotid plaques through deep learning and dimensionality reduction technology. In contrast, traditional clustering methods like hierarchical clustering are prone to low computational efficiency and difficult result visualization in large-sample high-dimensional data (15).

In addition, this study combined clinical practice and determined the optimal number of clusters as 5 through both the result of elbow method and silhouette score, ensuring the stability and clinical relevance of grouping. This process is more objective than preset rule-based grouping (such as dividing only by stenosis degree into mild/moderate/severe), consistent with the method of Blankenberg et al. (19) who identified 4 endotypes in subclinical atherosclerosis, both reflecting the advantages of data-driven typing.

### Plaque characteristics and metabolic features of different clusters: subgroup naming and key features

Based on the core characteristics of each subgroup, they can be named and summarized as follows:

The first subgroup, named Young Low-Risk Type, includes the youngest patients, at 64.17 ± 10.81 years, with the lowest NIHSS score of 8.74 ± 1.29. They have small plaque volume with a thickness of 2.14 ± 1.18 mm and high plaque stability, reflected by an unstable echo value of 0.55 ± 0.50, along with a mortality rate of only 2.9%. Their metabolic indicators, such as glycated hemoglobin at 7.20 ± 0.44%, and inflammatory levels, including a CRP of 17.75 ± 6.66 mg/L, are at moderate levels; this suggests the subgroup is characterized by early-stage stable plaques, and controlling traditional risk factors may effectively reduce associated risks (5).

The second subgroup, High Inflammation Large Plaque Type, has the highest NIHSS score of 14.81 ± 0.91, along with significantly elevated white blood cell counts (12.00 ± 0.73 × 10^9^/L) and creatine kinase levels (376.53 ± 27.36 U/L)—findings that point to severe inflammation and tissue damage (6). The severe inflammatory status in this subgroup is characterized by systemic inflammatory dysregulation, which can directly compromise endothelial integrity and vascular compliance, making the plaque more unstable and prone to detachment (20). Patients in this subgroup also have the largest plaques, with a thickness of 5.61 ± 8.21 mm and a length of 34.43 ± 19.15 mm, and extremely poor plaque stability (unstable echo value: 0.93 ± 0.26), which aligns with the high-risk features of Plaque-RADS grade 4 (12). Additionally, they have elevated lactic acid levels (3.19 ± 0.13 mmol/L) and hyponatremia (serum sodium: 135.85 ± 0.63 mmol/L), indicating tissue hypoxia and metabolic disorders, and the mortality rate here is 8.6%. This subgroup may represent a phenotype of severe systemic inflammation combined with large vulnerable plaques, which is associated with poor short-term prognosis and warrants close clinical surveillance (21).

The third subgroup, Elderly Stable Type, comprises the oldest patients, at 77.15 ± 8.16 years, but they have small plaque volume (thickness: 2.03 ± 1.16 mm), high plaque stability (unstable echo value: 0.48 ± 0.50), and the lowest inflammatory indicator levels with a CRP of 12.54 ± 6.24 mg/L. Although age is a strong predictor of atherosclerosis (4), their metabolic indicators, such as low-density lipoprotein at 2.55 ± 0.18 mmol/L, are well-controlled. The 8.4% mortality rate in this subgroup is likely related to other comorbidities associated with advanced age, rather than being directly caused by plaques.

The fourth subgroup, Chronic Inflammation Type, has the highest CRP level of 24.41 ± 12.86 mg/L, a sign of persistent inflammation (22). However, patients here have moderate plaque volume (thickness: 2.26 ± 1.21 mm) and a low stenosis rate of 1.88 ± 10.32%. Their metabolic indicators, such as glycated hemoglobin at 7.00 ± 0.39%, are slightly abnormal, and the 8.6% mortality rate may be linked to chronic inflammation accelerating plaque progression, suggesting that persistent inflammatory status may be an important therapeutic target for future intervention studies (9).

The fifth subgroup, High Stenosis High-Risk Type, has the highest stenosis rate of 15.84 ± 29.94%, and an unstable echo value of 0.78 ± 0.41, which suggests plaque vulnerability. Additionally, these patients have the highest glycated hemoglobin levels (7.22 ± 0.45%) and low-density lipoprotein levels (2.70 ± 0.25 mmol/L), with a mortality rate of 11.2%. This subgroup fits the “high stenosis + metabolic disorder” profile, consistent with Blinc et al.’s (10) conclusion that “plaque burden and metabolic abnormalities synergistically increase stroke risk”. These findings support that patients with combined severe stenosis and metabolic dysregulation may need intensified risk factor management and closer clinical follow-up (2).

The 30-day mortality rate of the fifth subgroup of Cluster 4, which stands at 11.2%, is notably higher than that of other subgroups, especially Cluster 0. This elevated mortality can be attributed to several core risk factors. Anatomically, Cluster 4 exhibits the highest stenosis rate of 15.84 ± 29.94%. Although most of the samples do not meet the severe stenosis standard of ≥70%, when combined with an unstable echo value of 0.78 ± 0.41, it conforms to the Plaque-RADS 3b/4a features (12), indicating a “low stenosis but high risk” phenotype (1). Plaques with such characteristics have an annual stroke incidence that can reach 6% (10). Metabolically, elevated glycated hemoglobin at 7.22% and low-density lipoprotein at 2.70 mmol/L contribute to plaque instability by promoting lipid deposition and oxidative stress (22). Hemodynamically, the decreased plasma viscosity of 1.37 ± 0.05 mPa·s may suggest blood stasis, which in turn increases the risk of thrombosis (23).

While this exploratory clustering analysis does not generate direct, cluster-specific therapeutic recommendations, the phenotypic characteristics of high-risk subgroups are consistent with established frameworks for secondary stroke prevention. Based on existing clinical evidence and consensus guidelines, optimized secondary prevention strategies may be prioritized in patients with elevated carotid plaque risk, as follows:

First, control of conventional vascular risk factors, including blood pressure management and regular aerobic physical activity, is essential to reduce the risk of recurrent stroke (3). For pharmacological management, guideline-based lipid management should be considered according to standard secondary prevention practice (2), and antiplatelet agents are routinely combined to mitigate thrombotic risk. Regarding revascularization assessment, evaluation for carotid endarterectomy (CEA) or carotid artery stenting (CAS) may be considered in patients with ≥50% symptomatic stenosis in combination with high-risk plaque features according to clinical practice criteria; in particular, intervention within 2 weeks of symptom onset has been shown to effectively reduce stroke risk (2). For metabolic management, optimization of glycemic control via dietary adjustment and appropriate glucose-lowering medications such as SGLT-2 inhibitors, optimization of glycemic control according to routine guideline-based care, helps attenuate the pathological drivers of plaque progression (21).

These evidence-based principles of secondary prevention provide a clinical context for the preliminary phenotypic stratification of short-term risk derived from our clustering analysis. Future prospective interventional studies are needed to verify whether management strategies tailored to specific phenotypic clusters can deliver additional clinical benefit.

### Advantages and limitations of this study

This study has significant advantages in methodological innovation and clinical value, but also has certain limitations.

The advantages of this study lie in the first simultaneous analysis of a full set of metabolic indicators and ultrasonic plaque features in a large sample, breaking through the limitation of “single dimension of imaging or metabolism” in traditional studies (17); meanwhile, combining *k*-means and UMAP to achieve objective typing, identifying subgroups easily ignored by traditional methods, providing new ideas for precise stratification (15). Moreover, the mortality differences across subgroups are associated with modifiable risk factors, providing clues for further exploration of individualized prevention and management strategies. Importantly, the addition of multivariable analysis further strengthened the clinical relevance of the findings by showing that prognostic differences persisted after adjustment for measured covariates, suggesting that the identified clusters may capture meaningful risk heterogeneity beyond unadjusted group differences. Moreover, the mortality differences across subgroups were associated with modifiable risk factors, providing clues for further exploration of individualized prevention and management strategies.

However, this study still has several limitations. First, the retrospective design may introduce selection bias, and the exclusion of asymptomatic plaque patients limits the generalizability of the findings. Second, because the study was designed around ultrasound and inflammatory/metabolic phenotyping, important stroke-related variables such as infarct characteristics, reperfusion therapy, and broader stroke-management factors were not incorporated into the clustering framework, which may limit broader prognostic interpretation. Although multivariable adjustment strengthened the prognostic analysis, residual confounding remains possible given the retrospective observational design and the likelihood of unmeasured or imperfectly measured baseline differences. Therefore, the prognostic results should be viewed as supportive evidence that the identified clusters have clinical relevance, rather than definitive proof that cluster membership independently captures underlying biological heterogeneity. A further limitation is that the robustness and reproducibility of the clustering solution were only moderately supported. Although the present analysis demonstrated distinct exploratory phenotypes, repeated-seed and bootstrap/subsampling ARI-based stability analyses showed that the five-cluster solution is reproducible under algorithmic and sampling perturbations but not perfectly invariant, and validation in independent external cohorts was not performed. Therefore, the identified clusters should be regarded as hypothesis-generating phenotypes rather than definitive patient subtypes, and their clinical interpretability should be considered tentative pending further confirmation. Only 30-day mortality was analyzed, and the long-term stroke recurrence risk of each subgroup was not evaluated, requiring verification by prospective cohorts; genomics or proteomics data were not included, making it difficult to conduct in-depth mechanism research. Future studies can further quantify plaque features through radiomics and more valuable deep learning methods to achieve a better model.

## Data Availability

The original contributions presented in the study are included in the article/Supplementary material, further inquiries can be directed to the corresponding author.
